# Returners and explorers dichotomy in human mobility

**DOI:** 10.1038/ncomms9166

**Published:** 2015-09-08

**Authors:** Luca Pappalardo, Filippo Simini, Salvatore Rinzivillo, Dino Pedreschi, Fosca Giannotti, Albert-László Barabási

**Affiliations:** 1 Institute of Information Science and Technology (ISTI), National Research Council (CNR), Via G. Moruzzi 1, 56124 Pisa, Italy; 2 Department of Computer Science, University of Pisa, Largo B. Pontecorvo 3, 56127 Pisa, Italy; 3 Center of Network Science, Central European University, Nador u. 9, 1051 Budapest, Hungary; 4 Institute of Physics, Budapest University of Technology and Economics, Budafoki u. 8, 1521 Budapest, Hungary; 5 Department of Engineering Mathematics, University of Bristol, Merchant Venturers Building, Woodland Road, BS8 1UB Bristol, UK; 6 CCNR and Physics Department, Northeastern University, 110 Forsyth Street, Boston, Massachusetts 02115, USA; 7 Department of Medicine, Brigham and Women's Hospital, Harvard Medical School, 25 Shattuck street, Boston, Massachusetts 56124, USA

## Abstract

The availability of massive digital traces of human whereabouts has offered a series of novel insights on the quantitative patterns characterizing human mobility. In particular, numerous recent studies have lead to an unexpected consensus: the considerable variability in the characteristic travelled distance of individuals coexists with a high degree of predictability of their future locations. Here we shed light on this surprising coexistence by systematically investigating the impact of recurrent mobility on the characteristic distance travelled by individuals. Using both mobile phone and GPS data, we discover the existence of two distinct classes of individuals: returners and explorers. As existing models of human mobility cannot explain the existence of these two classes, we develop more realistic models able to capture the empirical findings. Finally, we show that returners and explorers play a distinct quantifiable role in spreading phenomena and that a correlation exists between their mobility patterns and social interactions.

## Abstract (web summary)

Human mobility studies established that a high degree of predictability coexists with the variability of individual mobility. Here, the authors, by looking at mobile phone and GPS data, identify and model the emergence of two classes of mobility patterns in humans, returners and explorers.

## Graphical abstract

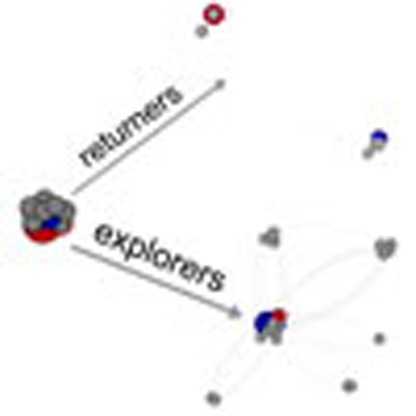

The availability of massive digital traces of human whereabouts has offered a series of novel insights on the quantitative patterns characterizing human mobility. Indeed, satellite-enabled global positioning systems (GPS) and mobile phone networks allow for sensing and collecting society-wide proxies of human mobility, like the GPS trajectories from vehicles and call detail records (CDR) from mobile phones. This broad social microscope has attracted scientists from diverse disciplines, from physics and network science^1^,^2^,^3^,^4^ to data mining^5^,^6^,^7^,^8^, and has fuelled advances from public health^9^,^10^,^11^,^12^,^13^,^14^ to transportation engineering^15^,^16^,^17^, urban planning^18^,^19^,^20^,^21^, official statistics^22^,^23^ and the design of smart cities^24^,^25^,^26^,^27^. All these studies document a stunning heterogeneity of human travel patterns that coexists with a high degree of predictability^28^,^29^: individuals exhibit a broad spectrum of mobility ranges while repeating daily schedules dictated by routine. Here we show that this seemingly conflicting coexistence of heterogeneity and predictability can be understood by quantifying the impact of recurring movements on mobility. To be specific, we analyse mobile call records and GPS tracks of private vehicles, allowing us to compare the overall mobility of an individual with her recurrent, or systematic, mobility. Two distinct mobility profiles emerge in both data sets: returners and explorers.

The characteristic distance travelled by returners, estimated by their radius of gyration^2^,^6^, is dominated by their recurrent movement between a few preferred locations. In contrast, recurrent mobility has only a vanishing contribution to the overall mobility of explorers, who have a tendency to wander between a larger number of different locations. We find that these two profiles are well-separated: individuals persistently belong to one or the other of these two classes. We show that current models of human mobility^4^ cannot account for these two classes of individuals and propose an improved model that can reproduce the mobility patterns of returners and explorers. Finally, we demonstrate that returners and explorers play different roles in spreading processes and that a strong correlation exists between the mobility behaviour of individuals and their social interactions.

## Results

### Data sets and measures

**Figure 1:**
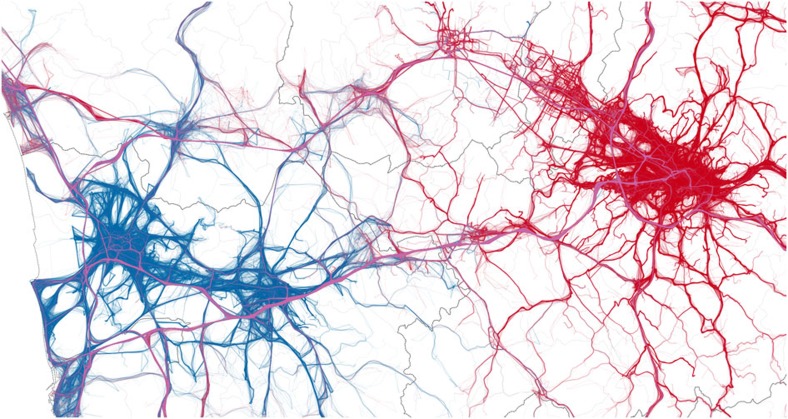
A visualization of the complexity of the explored mobility patterns. A fragment of the GPS trajectories used in our study, displaying trips originating in the metropolitan areas of Pisa (in blue) and Florence (red). This plain geo-referenced visualization of experimental data reveals the confrontation of two ‘competing' metropolitan areas. It also demonstrates the ability of Big Data to portray social complexity. This map has been generated through the QGIS software, available at http://www.qgis.org/en/site/.

Our first data source is an anonymized 3-month-long Global System for Mobile Communications (GSM) record collected by a European carrier for billing and operational purposes. It consists of CDR containing the calls of 67,000 individuals, selected from ∼3 million users provided that they visit more than 2 locations during the observational period and that their average call frequency *f* is ≥0.5 h^−1^ (see Supplementary Table 1, Supplementary Note 1). We reconstruct a user's movements based on the time-ordered list of cell phone towers from which a user made her calls^2^. Our second data source is a GPS data set that stores information about the trips of∼46,000 vehicles tracked during 1 month (May 2011), which passed through a 250 × 250 km square in central Italy. The visualization of the recorded trajectories demonstrates the complexity of explored mobility patterns (Fig. 1). We assign each origin and destination point of the obtained sub-trajectories to the corresponding Italian census cell, using information provided by the Italian National Institute of Statistics (ISTAT) (see Supplementary Table 2, Supplementary Note 2). We describe the movements of a vehicle by the time-ordered list of census cells where the vehicle stopped.

We use the total radius of gyration *r*_g_ defined as^2^,^6^:


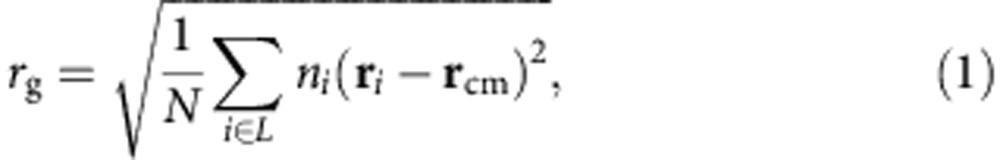


to characterize the typical distance travelled by an individual. Here *L* is the set of locations visited by the individual, **r**_*i*_ is a two-dimensional vector describing the geographic coordinates of location *i*; *n*_*i*_ is the visitation frequency or the total time spent by the individual in location *i*; 
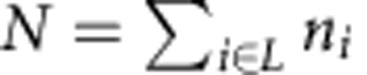
 is the total number of visits or time spent, and **r**_cm_ is the center of mass of the individual.

The most frequented location *L*_1_ is the place where an individual is found with the highest probability when stationary, most likely her home. In general, the importance of each location *L*_*k*_ to an individual is defined by its rank, where *L*_*k*_ is the *k*-th most frequented location (Supplementary Note 3, Supplementary Fig. 1).

### Returners and explorers

To understand how the *k*-th most frequented locations of an individual determine the characteristic distance travelled by her, we define the *k*-radius of gyration 
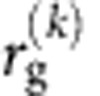
.


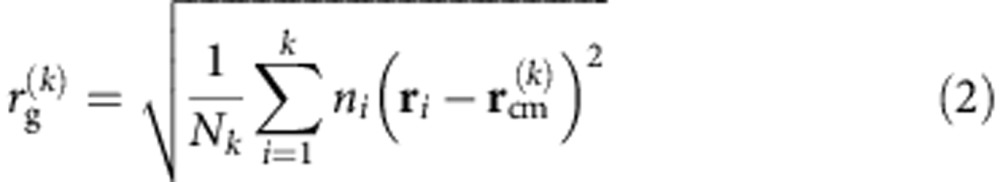


as the radius of gyration computed over the *k*-th most frequented locations *L*_1_,…,*L*_*k*_; 
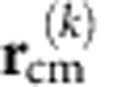
 is the centre of mass computed on the *k-*th most frequented locations (Supplementary Note 7, Supplementary Figs 8 and 9); *N*_*k*_ is the sum of the weights assigned to the *k-*th most frequented locations (
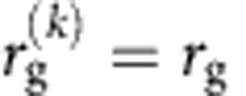
 if *k*≥*N*). Thus, 
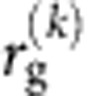
 represents the mobility range restricted to the *k-*th most frequented locations. For example, if an individual's 
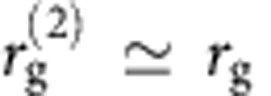
, then her characteristic travelled distance is dominated by the two most frequented locations. Conversely, if the 
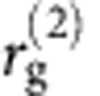
 is much smaller than the total *r*_g_ the two most frequented locations do not offer an accurate characterization of the individual's travel pattern and we need to consider more locations.

**Figure 2:**
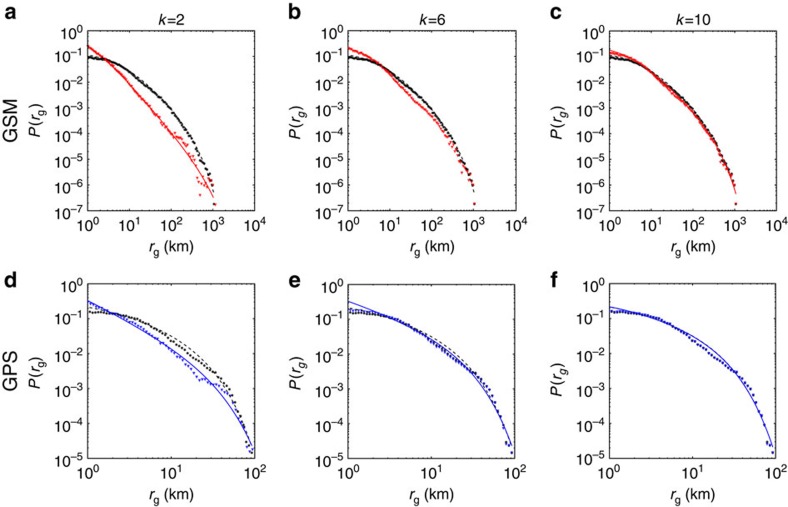
The distributions of k-radii and total radii. The distributions of total *r*_g_ and 
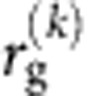
, with *k*=2, 6, 10 for the GSM data (**a**–**c**) and GPS data (**d**–**f**). Black circles indicate the total *r*_g_, red and blue triangles indicate the 
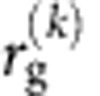
 for the GSM and GPS data, respectively. All distributions are approximated by a truncated power law 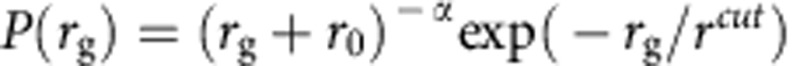; the dashed black line represents a truncated power-law fit of the total *r*_g_, the red and blue solid lines represent a truncated power-law fit of 
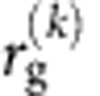
 for the GSM and GPS data, respectively. Table 1 shows the fitting parameters of the truncated power laws.

To investigate the role of the *k-*th most frequented locations for an individual's mobility pattern, we compare the probability distributions of total *r*_g_ and 
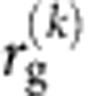
 for *k*=2,…,10 for the GSM and the GPS data (Fig. 2). All curves are long-tailed, indicating that most individuals cover small distances, but a few travel regularly over hundreds of kilometers (heterogeneity). We fit the distributions using the truncated power law^2^,^6^ 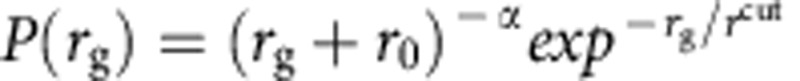 (Fig. 2), finding two significant differences. First, the exponent *α* of the distribution of *k*-radii is significantly higher than the exponent of the total *r*_g_ (see Table 1). Second, the exponential cutoff parameter *r*^cut^ is larger for small *k* (see Table 1). Obviously, as *k* increases the 
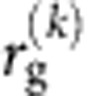
 curve approaches the total *r*_g_ distribution.

**Figure 3:**
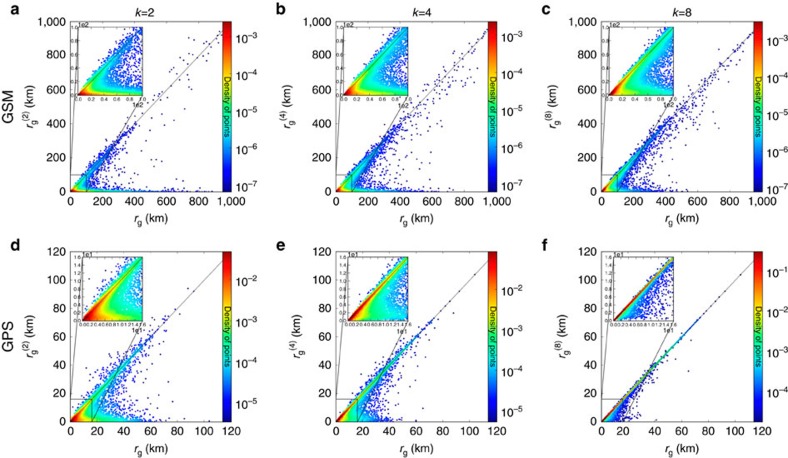
The correlation between recurrent and overall mobility. The scatter plots represent the correlation between total *r*_g_ and 
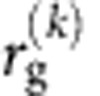
 for *k*=2, 4, 8 in the GSM data set (**a–c**) and the GPS data set (**d–f**). Each point is coloured from blue to red, indicating the density of points in the corresponding region. Most of the points gather around the *x*-axis, the diagonal and the origin. The insets magnify the origin of the plot to [0, 100 km] for GSM and [0, 16 km] for GPS, demonstrating that the split emerges for smaller radii as well. As *k* increases explorers become returners. This transition is faster in the GPS case, consistent with the fact that the vehicle mobility represents a subset of trips and visited locations.

The correlation between total radius and *k*-radius of gyration allows us to quantify the degree of similarity between overall and recurrent mobility. Figure 3 compares total *r*_g_ and 
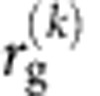
 of each individual for *k*=2, 4, 8, indicating that the population splits into two distinct classes. The data points concentrated around the diagonal correspond to individuals whose total *r*_g_ is comparable to 
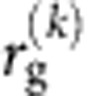
, indicating that their characteristic travelled distance is dominated by their *k-*th most frequented locations. We call them *k*-returners, as their mobility range is well-approximated by their *k-*th most frequented locations. The points concentrated around the abscissa correspond to individuals whose 
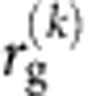
 is considerably smaller than total *r*_g_, indicating that we cannot reduce their mobility to *k* locations; we call them *k*-explorers. For example, the characteristic travelled distance of a two-returner is mainly determined by the two most frequented locations, typically corresponding to her home and work. In contrast, a two-explorer travels recurrently between many different locations.

The separation between the two classes is especially clear for high radii of gyration, as for high total *r*_g_ we find very few points between the diagonal and the abscissa in Fig. 3. Yet, as the insets show, the split into the two classes is valid for smaller total *r*_g_ as well. The number of *k*-returners increases with *k* (Supplementary Fig. 3), and when *k* equals the total number of visited locations each individual becomes a returner. Note that while explorers gradually become returners as *k* increases, the opposite process is extremely rare (see Supplementary Note 8, Supplementary Fig. 10). The partition of individuals into returners or explorers observed in both the GSM and the GPS data is not due to confounding variables like the heterogeneity of the number of calls or the demography of the municipality of residence (see Supplementary Note 6, Supplementary Fig. 7).

We develop three algorithms to split the population into *k*-returners and *k*-explorers: the bisector method classifies an individual as a *k*-returner if 
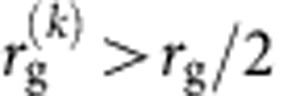
 or a *k*-explorer otherwise; a support vector machine classifier and the expectation-maximization clustering algorithm^30^ extract the two patterns from the population (see Supplementary Note 4, Supplementary Fig. 2). The three methods produce similar results, indicating that the two classes are clearly separated and well-defined. Consequently, in the following we use the simpler bisector method to split the population into *k*-returners and *k*-explorers.

**Figure 4:**
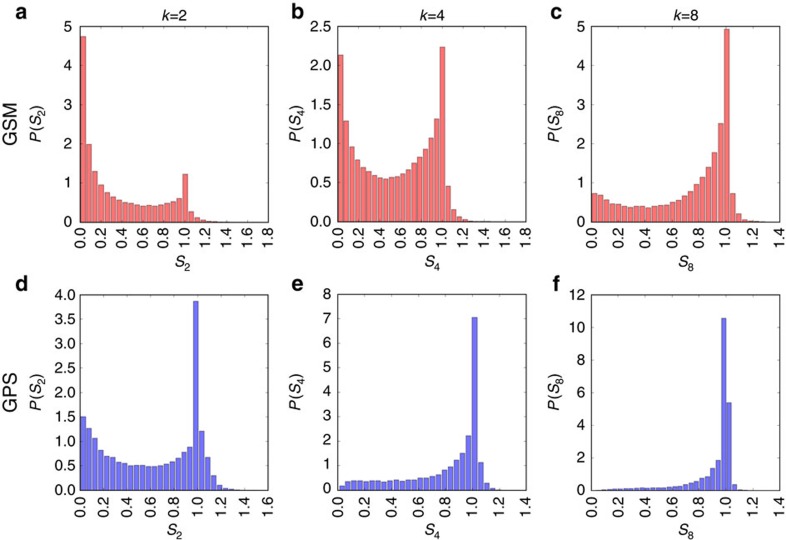
The ratio between recurrent and overall mobility. The distribution *P*(*s*_*k*_) of the ratio 
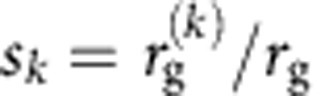
 measured on the GSM data for *k*=2, 4, 8 (**a**–**c**). The peak at *s*_*k*_=0 corresponds to explorers, while the *s*_*k*_=1 peak corresponds to returners. For small *k* in the GSM data, *k*-explorers are more numerous than *k*-returners. As *k* increases the number of *k*-returners increases and overcomes the number of *k*-explorers. A balance in the population is reached at *k*=4. (**d**–**f**) The *P*(*s*_*k*_) for the GPS data. We again observe two peaks, but the *k*-returners peak, *s*_*k*_=1, dominates for all *k*≥2.

The ratio 
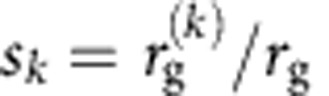
 measures the impact of an individual's recurrent mobility on her overall mobility: the higher the ratio the higher is the weight of the top *k* locations in the trajectories of an individual. Figure 4 shows the probability distribution of the *s*_*k*_ ratio for different *k*. We observe two peaks: the peak located at *s*_*k*_=0 corresponds to *k*-explorers, whose *k*-radius is significantly smaller than the total *r*_g_; the peak at *s*_*k*_=1 corresponds to the *k*-returners, individuals whose 
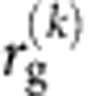
 is very similar to the total *r*_g_. Note that only for a few individuals *s*_*k*_>1 (that is 
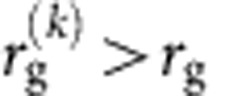
), suggesting that for the great majority of the individuals the *k-*th most frequented locations are on average closer to the centre of mass than their remaining less frequented locations (see Supplementary Note 9, Supplementary Fig. 11). By increasing *k*, the *k*-explorers gradually become *k*-returners, causing the explorers and returners peaks to decrease and increase, respectively. The population reaches a balance of *k*-returners and *k*-explorers for *k*=4 for GSM. In the GPS data, regardless of *k*, we always have more *k*-returners than *k*-explorers. A possible reason is that GPS data only contains trips made by private vehicles, hence missing long distance trip locations less frequented by a particular individual, reached by train or plane. These trips increase the total *r*_g_ without affecting the 
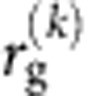
. Neglecting these trips results in a lower estimate of an individual's total *r*_g_, increasing the chance to classify her as a returner.

**Figure 5:**
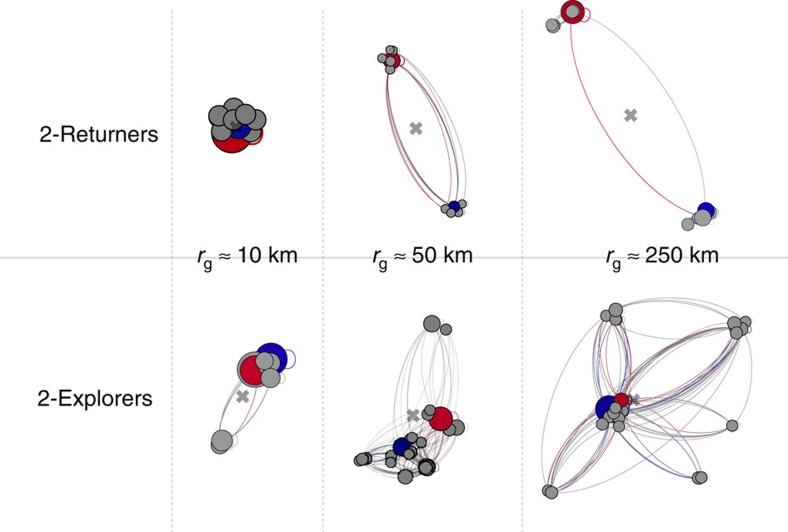
The individual mobility networks of returners and explorers. The mobility networks of returners and explorers for *k*=2. Nodes (circles) indicate the geographic locations visited by the individual, and each link denotes a travel observed between two locations. When the total *r*_g_ is small, the two most important locations (red and blue) are close to each other for both two-explorers and two-returners. As the total radius increases the behaviour of two-returners and two-explorers starts to differ; for two-returners, the two most important locations move away from each other; for two-explorers, they stay close and other clusters of locations emerge far from the centre of mass (the grey cross).

Returners and explorers are also characterized by a different spatial distribution of the visited locations. Figure 5 shows some representative examples of individual mobility networks^3^,^31^ of two-returners and two-explorers with different total *r*_g_. For both profiles, the visited locations tend to group in dense clusters with few outliers (see Supplementary Note 10, Supplementary Fig. 12). For two-returners the distance between the two most frequented locations is proportional to the total *r*_g_; in contrast, for two-explorers the distance between the two most frequented locations is much smaller than the total *r*_g_, whose magnitude is mostly determined by less frequented locations far from the centre of mass. Indeed, the distance between the two most frequented locations grows with total *r*_g_ more rapidly for returners than explorers (see Supplementary Note 5, Supplementary Fig. 4), and while the locations visited by two-returners are clustered around their two most frequented locations those visited by two-explorers are more spread out (see Supplementary Note 5, Supplementary Figs 5 and 6). The higher the total radius of gyration, the more obvious is the difference between the two profiles.

### Models

**Figure 6:**
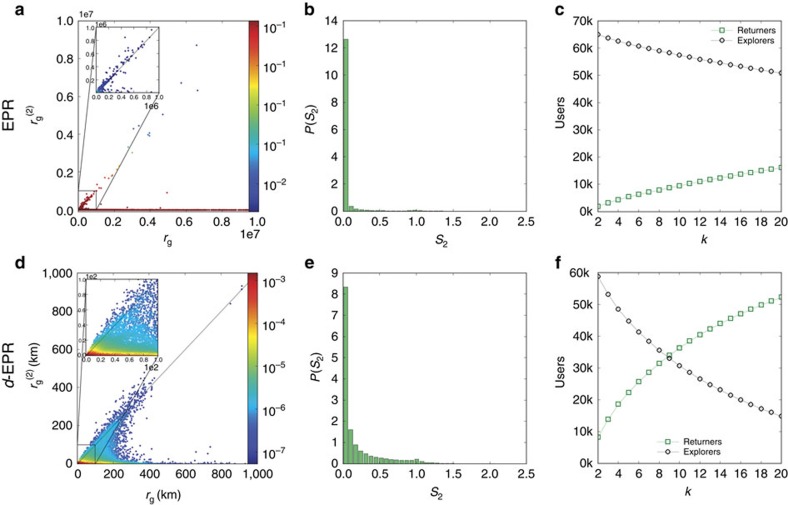
EPR model predictions. (**a**,**b**) The prediction of the EPR model for *k*=2. We find that two-explorers dominate the population of synthetic individuals and the balance in the population is reached only for *k*=60, in contrast with *k*=4 in the empirical data. (**d**,**e**) The results of the *d*-EPR model for *k*=2. In this case, the two-explorers continue to dominate the population, although the balance is reached at lower values of *k*=9, coming closer to empirical data. The insets in **a,d** magnify the plot at smaller values of the radii of gyration. Plots (**c**,**f**) show how the number of *k*-returners and *k*-explorers changes with *k* for EPR model and *d*-EPR model, respectively.

We compare our findings with the results produced by the exploration and preferential return (EPR) individual mobility model^4^, a state-of-the-art model that accurately captures the visitation frequency of locations, the distribution of the radius of gyration across the population and its growth with time (ultraslow diffusion). The model incorporates two competing mechanisms, the exploration of new locations and the return to previously visited locations. We use the EPR model to simulate the mobility of 67,000 synthetic individuals (see Box 1, and Supplementary Notes 11 and 12) and computed for each synthetic individual the total *r*_g_ and 
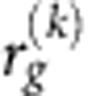
. As shown in Fig. 6a although for *k=*2 there is a weak tendency for points to gather around the diagonal, the empirically observed split into returners and explorers is absent from the model trajectories (see Supplementary Fig. 13). The difference between the empirical and synthetic data is especially clear when we explore *P*(*s*_*k*_) (Fig. 6b versus Fig. 4). For small *k*, in the model *k*-explorers (with the ratio *s*_*k*_≈0) dominate the population. For *k*≈60, we have the perfect balance between *k*-returners and *k*-explorers as for the GSM data set for *k*=4 (Fig. 4b, Supplementary Fig. 14). Thus, the EPR model overestimates by more than an order of magnitude the number of locations needed to accurately estimate the total radius of gyration. Contrarily to the empirical results, in the EPR model there is no significant correlation between total *r*_g_ and the sum of the distances of the *k-*th most visited locations (Pearson correlation coefficient is close to zero), neither for *k*-returners nor for *k*-explorers (see Supplementary Fig. 15a,b).

The observed discrepancies between the empirical data and the EPR model could arise from the fact that in the model individuals can travel arbitrarily large distances, increasing their total *r*_g_ with each jump. To correct for this limitation, we propose the *d*-EPR model, in which an individual selects a new location to visit depending on both its distance from the current position, as well as its relevance measured as the overall number of calls placed by all individuals from that location. We use the gravity model^32^,^33^ to assign the probability of a trip between any two locations, which automatically constrains individuals within the country's boundaries (see Supplementary Notes 11, 13 and 14, Supplementary Fig. 16). This modification is justified by the accuracy of the gravity model to estimate origin-destination matrices at the country level^34^,^35^,^36^,^37^. The obtained *d*-EPR model generates trajectories that are in much better agreement with the empirical data: the balance between *k*-returners and *k*-explorers in the population is reached at *k*≈9, in contrast with *k*≈60 in the original EPR model (Fig. 6f), closer to *k*=4 in GSM and *k*=2 in GPS (Fig. 3). Consequently, the correlation plot of 
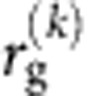
 versus total *r*_g_ displays the empirically observed split into returners and explorers (Fig. 3 even at *k*=2, Fig. 6d). The correlation between total *r*_g_ and the distance between the most visited locations is much higher than in the original EPR model and closer to the values of GSM and GPS data (see Supplementary Fig. 15e,f).

Hence, the *d*-EPR model of human mobility reproduces the key features of the aggregated mobility patterns in a confined geographical space, accounting for the two classes of individuals, returners and explorers. The mechanism underlying the model can be easily understood: when a traveller returns, she is attracted to previously visited places with a force that depends on the relevance of such places at an individual level. In contrast when a traveller explores, she is attracted to new places with a force that depends on the relevance of such places at a collective level.

### The relevance of returners and explorers dichotomy

Our findings are particularly relevant in two contexts: the geographical spreading of epidemics and social interactions. The geographical spreading of an epidemic is a direct consequence of individuals' movements^9^,^12^,^38^,^39^. From the ‘patient zero' (that is, the first infected individual), the virus is passed on to individuals who come into contact with them, contributing to the rapid growth of the epidemic. Obviously, the wider the range of mobility, the faster will the virus diffuse over the population. The question is, how does the presence of the two mobility profiles uncovered above affect the spreading pattern? To test this, we split the mobility history of an individual into time periods, and captured the trajectory's reach up to time *t* using three measures: (i) the number of locations visited; (ii) the area covered; and (iii) the total radius of gyration *r*_g_(*t*). We observe that the trajectory of explorers is distributed over a larger territory, as they visit more locations, cover a larger geographic area and have a higher *r*_g_(*t*) with respect to returners. This pattern applies both for GSM and GPS data (see Supplementary Note 15, Supplementary Fig. 17). We also assess the different role the returners and explorers play in diffusion and spreading processes by considering the global mobility networks generated by individual mobility. The global mobility network is a graph whose nodes are locations and edges indicate the existence of at least one trip between two locations. To be specific, we focus on Tuscany, estimating the mobility of each individual through the GPS data and the number of residents in the locations through the official census cells provided by the ISTAT. We build 10 global mobility networks considering the trips of 10,000 randomly selected individuals, choosing different proportions of two-returners and two-explorers (0%, 10%,…, 100% of two-explorers in the random population). For each network, we compute the global invasion threshold R_*_ under the assumption of a diffusion dynamics with large subpopulations and a low reproductive number (that is, close to the subpopulation epidemic threshold)^40^ (Supplementary Note 16). In a metapopulation network, an epidemic can spread and invade the system only if R_*_>1, and this global invasion threshold is affected by the topological fluctuations of the network's degree: the larger the degree heterogeneity, the higher the R_*_ and therefore the higher is the chance that the epidemic will globally invade the metapopulation. We compute each of the 10 networks 1,000 times, randomly choosing 10,000 individuals with different proportion of two-returners and two-explorers, and obtaining 1,000 values for the invasion threshold for each network (Supplementary Note 16, Supplementary Fig. 18). We observe that the mean diffusion invasion threshold increases with the fraction of explorers in the random population. Although more refined metapopulation infection models are needed to provide accurate estimates of invasion probabilities, our analysis reveals a clear distinction between the diffusion properties of the returners and explorers' mobility networks.

**Figure 7:**
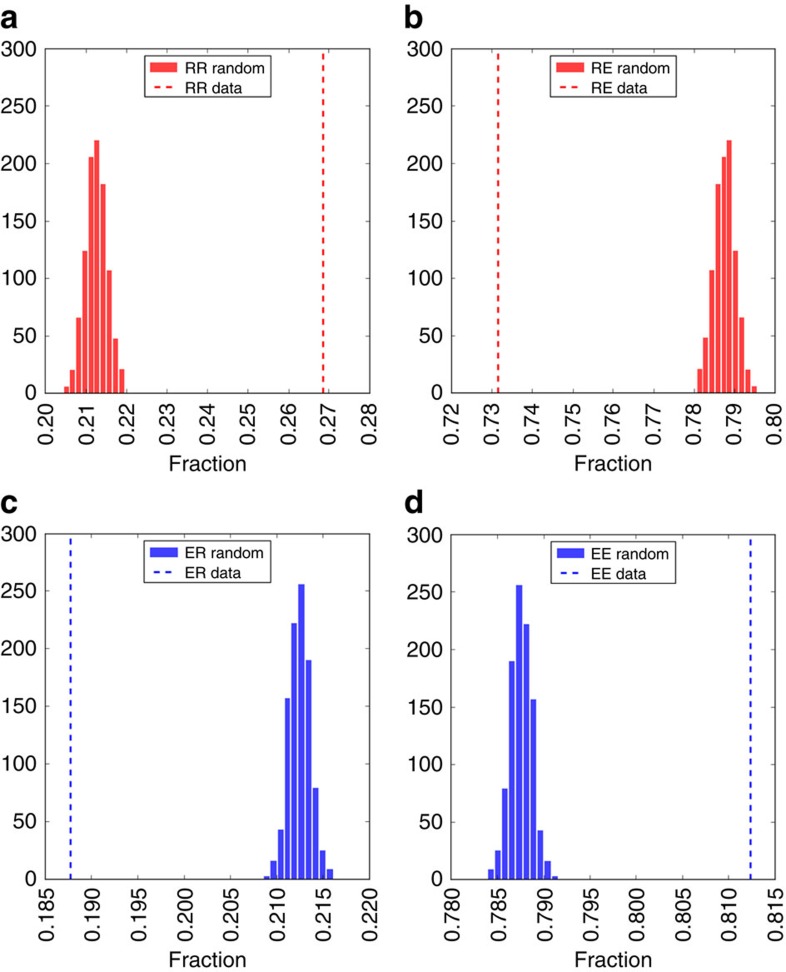
Best friend interaction patterns of returners and explorers. The histograms indicate the distributions of the fraction of two-returners whose best friend (that is, the most called contact) is a two-returner (*RR*) (**a**) or a two-explorer (*RE*) (**b**), and two-explorers whose best friend is a two-returner (*ER*) (**c**) or two-explorer (*EE*) (**d**), obtained from 100,000 randomized experiments where we randomly reassign each individual's best friend. The dashed line indicates the real fraction of two-returners (two-explorers) whose best friend is a two-returner (two-explorer). We observe that individuals that communicate with each other are more likely to belong to the same mobility group than by chance.

**Figure 8:**
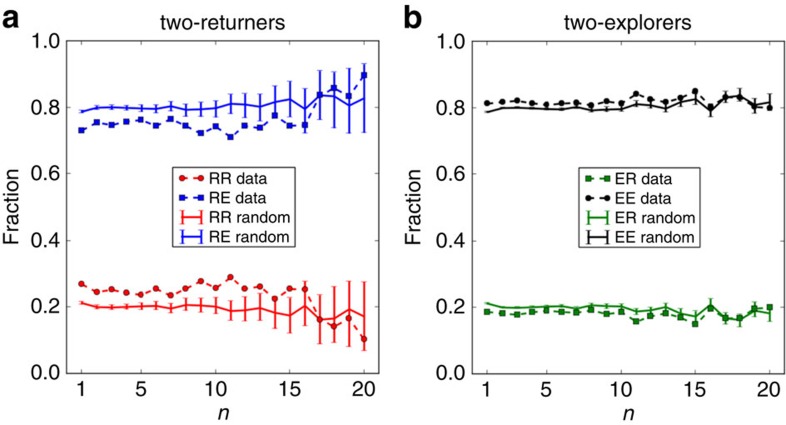
Interaction patterns of returners and explorers versus the rank of friendship. (**a**) The fraction of two-returners having a two-returner (*RR*) or a two-explorer (*RE*) as *n*-th best friend as *n* increases. (**b**) The fraction of two-explorers having a two-returner (*ER*) or a two-explorer (*EE*) as *n*-th best friend as *n* increases. We observe that the observed the fractions are significantly higher (*RR* and *EE*) or significantly lower (*ER* and *RE*) than those obtained by chance for all *n* up to 15.

Recent advances in characterizing the signature^41^ or strategies^42^ of social interactions and the possibility to exploit the information on an individual's social ties to predict her future locations^43^,^44^ demonstrate a strong connection between social interactions and human mobility patterns. Here we bring a further contribution by showing that individuals of the two profiles, returners and explorers, tend to engage in social interactions preferably with individuals of the same profile. In other words, individuals who communicate with each other are more likely to belong to the same mobility group than by chance. In particular, we find that the fraction of two-returners whose ‘best friend' (that is, the most called contact) is also a two-returner is *RR*≈0.27. We compare this figure with the highest fraction of two-returners best friends obtained from 100,000 randomized experiments where we randomly reassign each individual's best friend, obtaining *RR*_rand_≈0.21, resulting in a highly significant *P* value (<10^−5^), as shown in Fig. 7a,b. The same applies to two-explorers (*EE*≈0.81, *EE*_rand_≈0.78), as shown in Fig. 7c,d. As we consider the *n*-th most called contact and compare the fraction of individuals with the *n*-th best friend in the same mobility group, we find that the observed fractions are significantly higher than those obtained by chance for all *n* up to 15, as shown in Fig. 8. Our findings reveal the existence of a strong correlation between the mobility behaviour of individuals and their social relationships, although further experiments are needed to understand whether this can be interpreted as a homophily or influence effect.

## Discussion

Here we report the existence of two distinct profiles characterizing human mobility: returners and explorers. Returners limit much of their mobility to a few locations, hence their recurrent and overall mobility are comparable. In contrast, the mobility of explorers cannot be reduced to few locations. These patterns cannot be explained by the EPR model of human mobility, unable to distinguish returners from explorers. We show that by incorporating a gravity model into the EPR mechanism, we can recover the two classes, the obtained extended model coming closer to the empirical observations characterizing the two profiles. The returner/explorer dichotomy has a strong impact on spreading and social interactions. We show that explorers and returners play different roles in the disease spreading and that they tend to engage in social interactions with individuals with similar mobility profiles. The emerging profiles of returners and explorers offer another step towards deriving accurate models of human mobility, capable of generating realistic simulations, predictions and what-if reasoning in context such as energy consumption, gas emission and urban planning^45^.

## Additional information

**How to cite this article:** Pappalardo, L. *et al.* Returners and explorers dichotomy in human mobility. *Nat. Commun.* 6:8166 doi: 10.1038/ncomms9166 (2015).

## Supplementary Material

Supplementary Information Supplementary Figures 1-18, Supplementary Tables 1-2, Supplementary Notes 1-16 and Supplementary References

## Figures and Tables

**Table 1:** Fitting parameters of truncated power laws.

	**GSM**			**GPS**		
	***r***_**0**_	***α***	***r***^**cut**^	***r***_**0**_	***α***	***r***^**cut**^
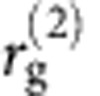	0.82	1.89	691.03	0.07	1.25	22.92
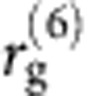	1.44	1.61	308.47	0.13	0.9	16.36
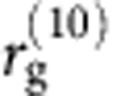	2.88	1.6	275.22	0.91	0.76	14.56
*r*_g_	5.5	1.6	250.11	0.96	0.75	14.44

The parameters of the fitted truncated power laws for 
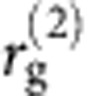
, 
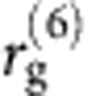
, 
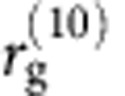
 and total 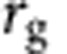 for GSM and GPS data.

## Box

**The *d*-EPR model**. We place each of the 67,000 GSM users in their most frequented location *L*_1_ (GSM cell phone towers). For each individual, we repeat the following steps for 3 months:

**Waiting time choice.** We extract a waiting time *Δt* from the distribution *P*(*Δt*)∼*Δt*^*−*1*−β*^ exp^(*−Δt/τ*)^ with *β=*0.8 and *τ*=17 h, as measured in ref. 4.

**Action selection**. With probability *P*_new_=*ρS*^−γ^, where *S* is the number of distinct locations previously visited, *ρ*=0.6 and *γ*=0.21 (ref. 4), the individual visits a new location (step 3), otherwise she returns to a previously visited location (step 4).

**Exploration phase.** If the individual who is currently in location *i* explores a new location, then the new location *j*≠*i* is selected according to the gravity model^32^,^33^ with probability , where  is the location's relevance, that is, the total number of calls placed by all individuals from location *i*(*j*), *r*_*ij*_ is the geographic distance between *i* and *j*, and  is a normalization constant. The number of distinct locations visited, *S*, is increased by 1.

**Return phase.** If the individual returns to a previously visited location, it is chosen with probability proportional to the number of visits to that location.
