# Supplementary material for: Returners and explorers dichotomy in human mobility
Source: Nat Commun. 2015 Sep 8;6:8166. doi: 10.1038/ncomms9166 (PMC4569739; doi:10.1038/ncomms9166)
Supplement: Supplementary Information — Supplementary Figures 1-18, Supplementary Tables 1-2, Supplementary Notes 1-16 and Supplementary References [file ncomms9166-s1.pdf]

1 Supplementary Figures

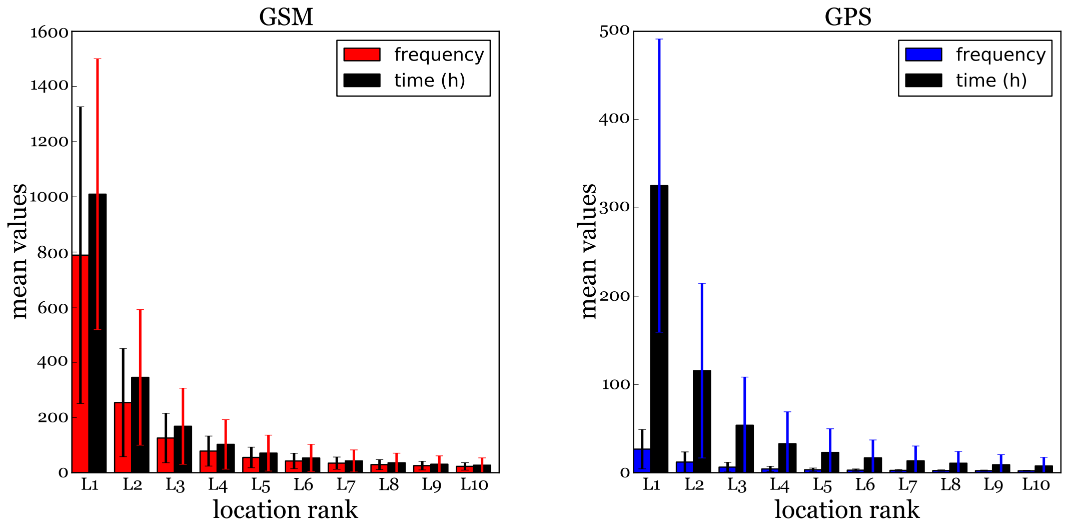

Supplementary Figure 1: **The distribution of frequency and dwell time of the most frequented locations.** Bar charts of the mean frequency and mean dwell time (in hours) of the users in the ten most frequent locations, for the GSM (left) and the GPS (right) datasets. Whiskers indicate the standard deviation of frequencies and times.

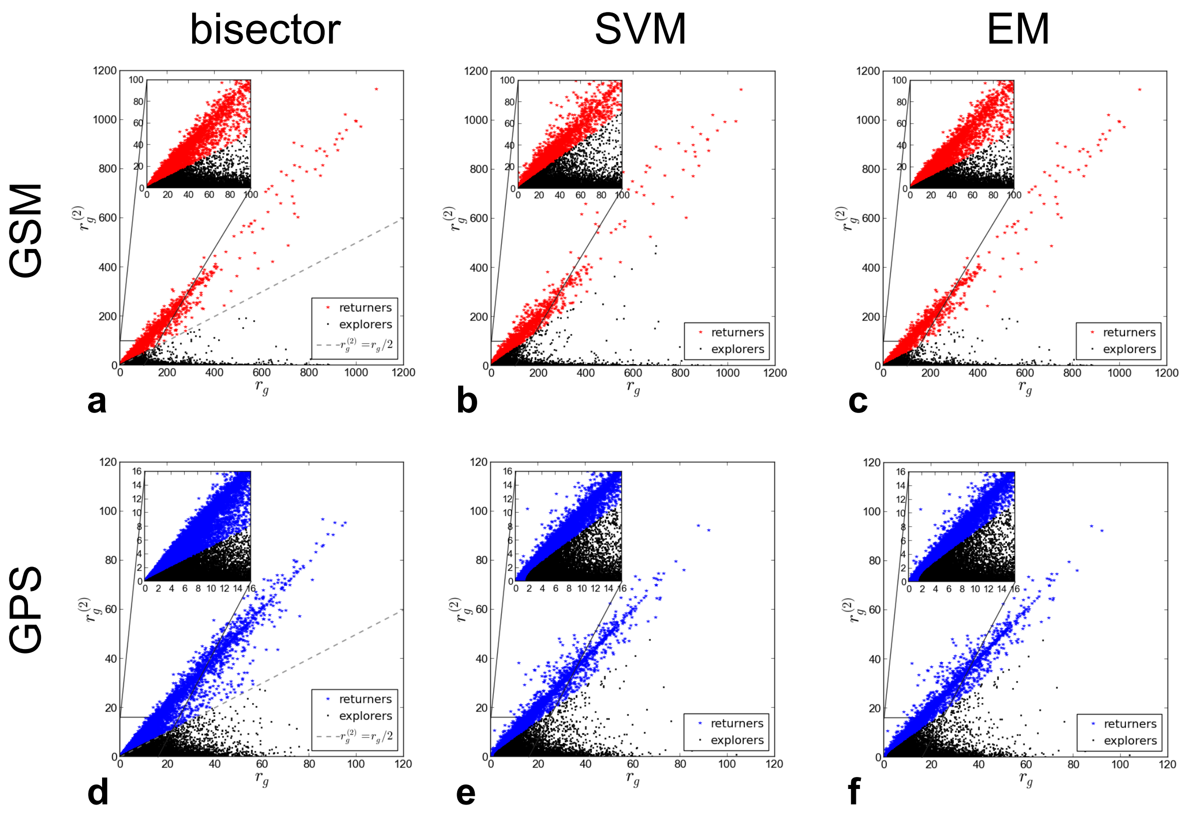

Supplementary Figure 2: **Classification of returners and explorers**. Split of the population in 2-returners and 2-explorers according to the the three split methods on GSM data (**a, b, c**) and GPS data (**d, e, f**).

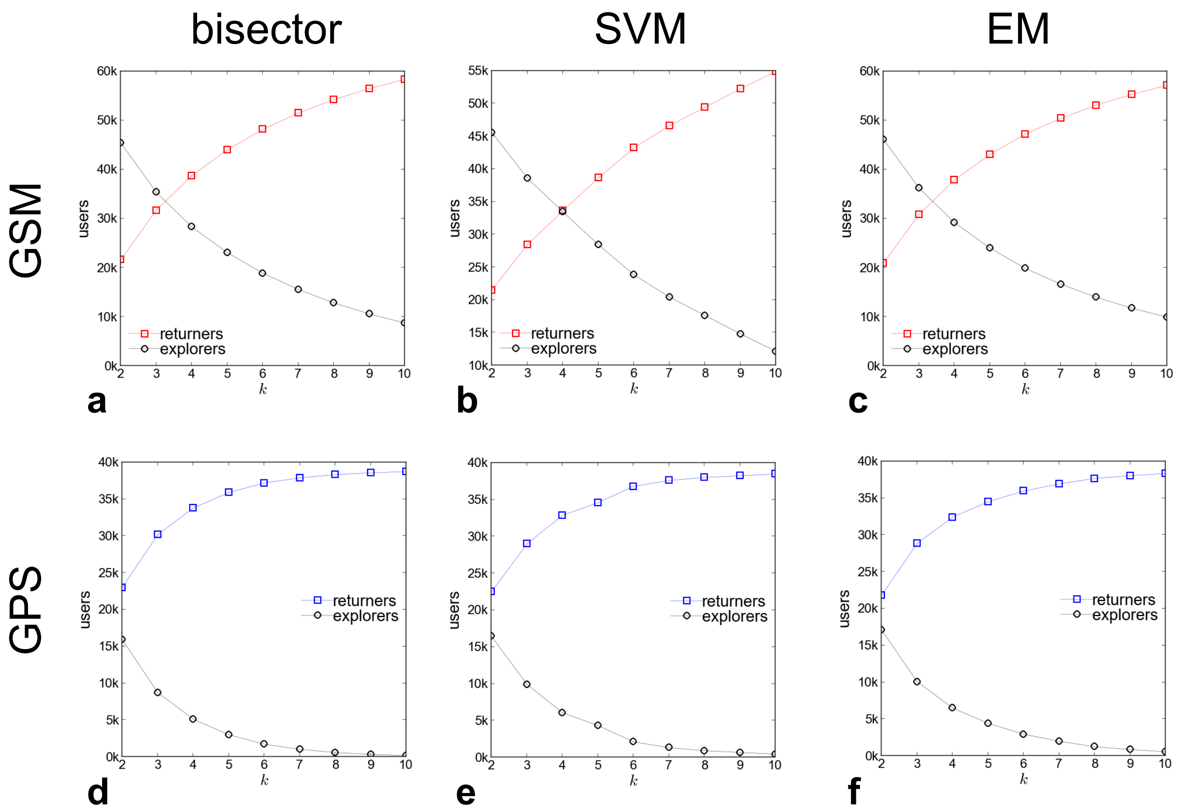

Supplementary Figure 3: **Number of returners and explorers as k increases.** Number of k-returners and k-explorers in the population with $k = 2, \ldots, 10$ for GSM data (**a, b, c**) and GPS data (**d, e, f**) according to the three spit methods. In GSM data a balance of the two profiles is reached at $k = 4$, while in GPS data k-returners are immediately more numerous than k-explorers.

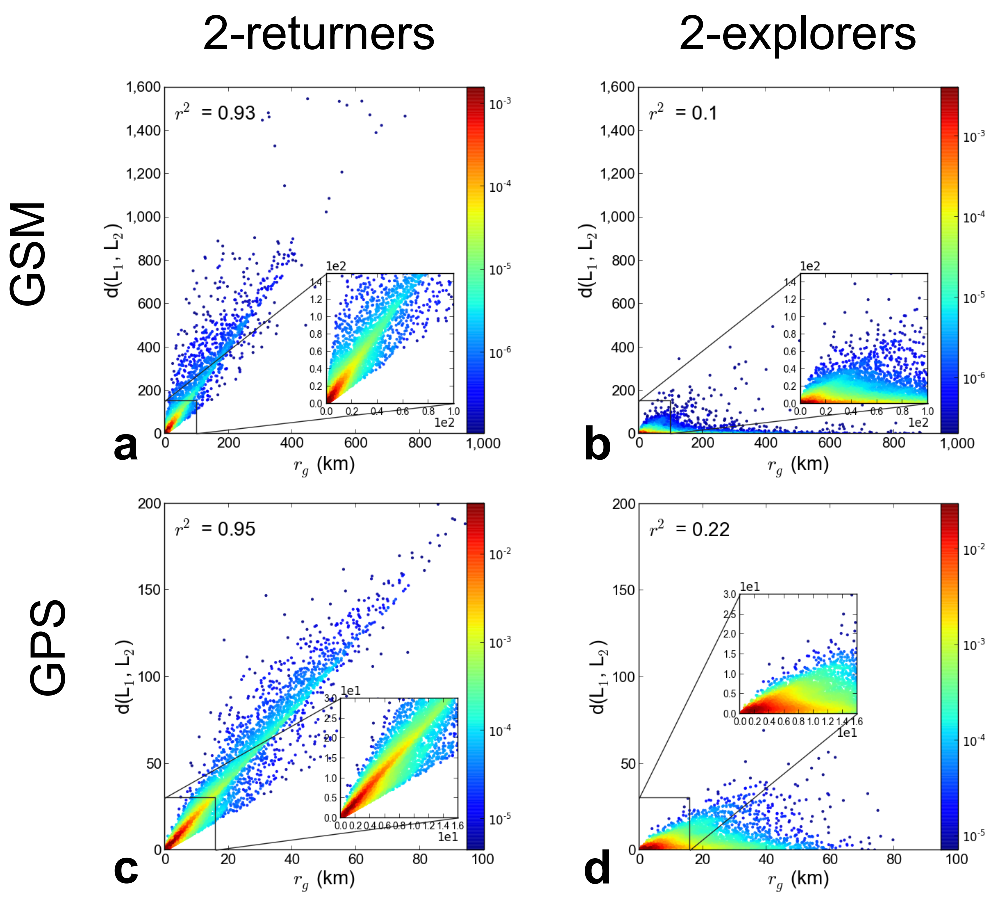

Supplementary Figure 4: **The correlation between total radius and distance of the most frequented locations.** Scatterplots of r_g versus the distance between the two most frequent locations $dist(L_1, L_2)$ for 2-returners and 2-explorers, for GSM data (**a, b**) and GPS data (**c, d**). The correlation is much stronger for 2-returners than 2-explorers, as we can see from the values of the coefficient of determination r^2.

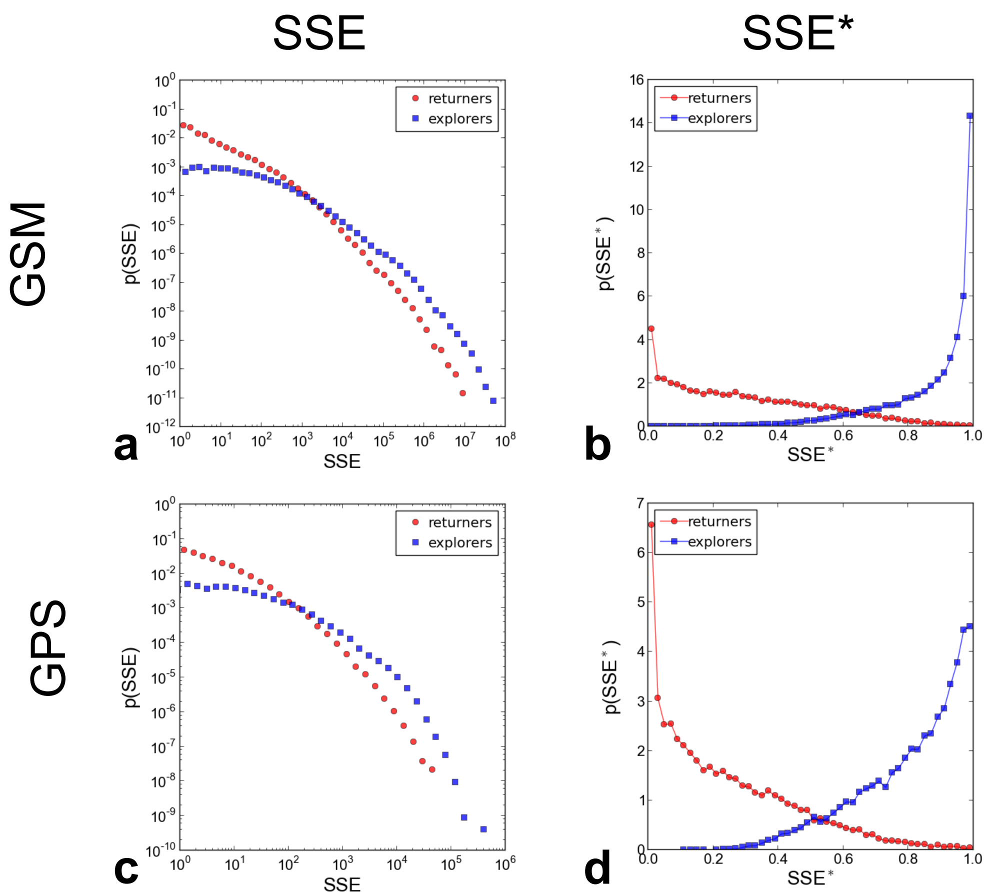

Supplementary Figure 5: **The distributions of SSE and SSE*.** Distribution of SSE for GSM data (**a**) and GPS data (**b**) separately for 2-returners and 2-explorers. Distribution of SSE* for GSM data (**c**) and GPS data (**d**) separately for 2-returners and 2-explorers.

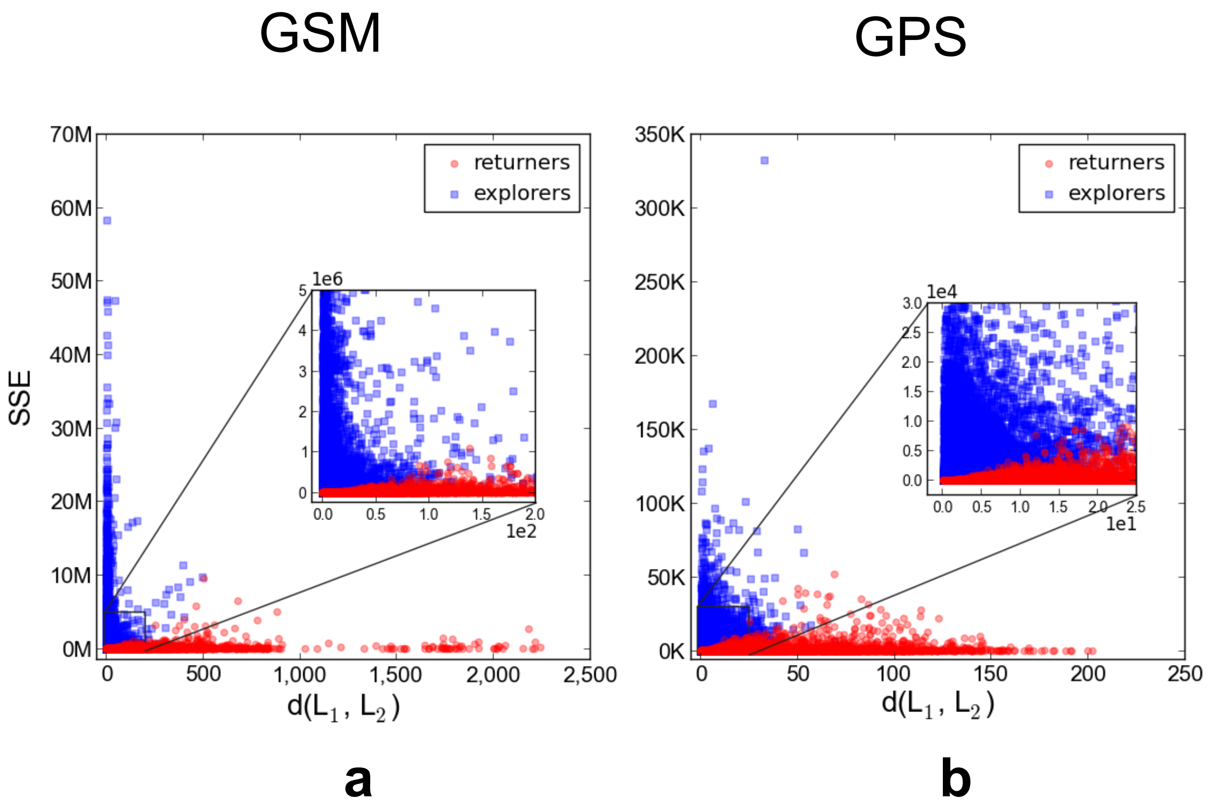

Supplementary Figure 6: **The correlation between the distance of most frequented locations and SSE.** Scatterplot of distance $d(L_1, L_2)$ and SSE separately for 2-returners and 2-explorers, for GSM data (**a**) and GPS data (**b**).

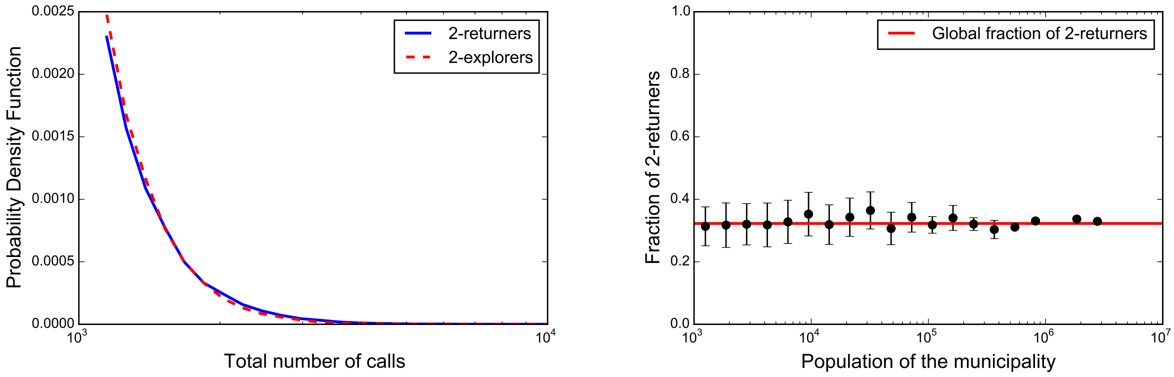

Supplementary Figure 7: **The role of call activity and demographic variables.** *(Left)* Distribution of total number of calls made by 2-returners (blue solid curve) and 2-explorers (red dashed curve). We observe that the curves are very similar excluding a possible bias due to heterogeneous call frequencies. *(Right)* The red solid curve represents the global fraction of 2-returners in the population (GSM data), the black error bars represents the distribution (mean and the standard deviation) of the fraction of 2-returners living in municipalities with a given population. We observe that the fraction of 2-returners is independent of the population of the municipality and compatible with the overall fraction of 2-returners in the country.

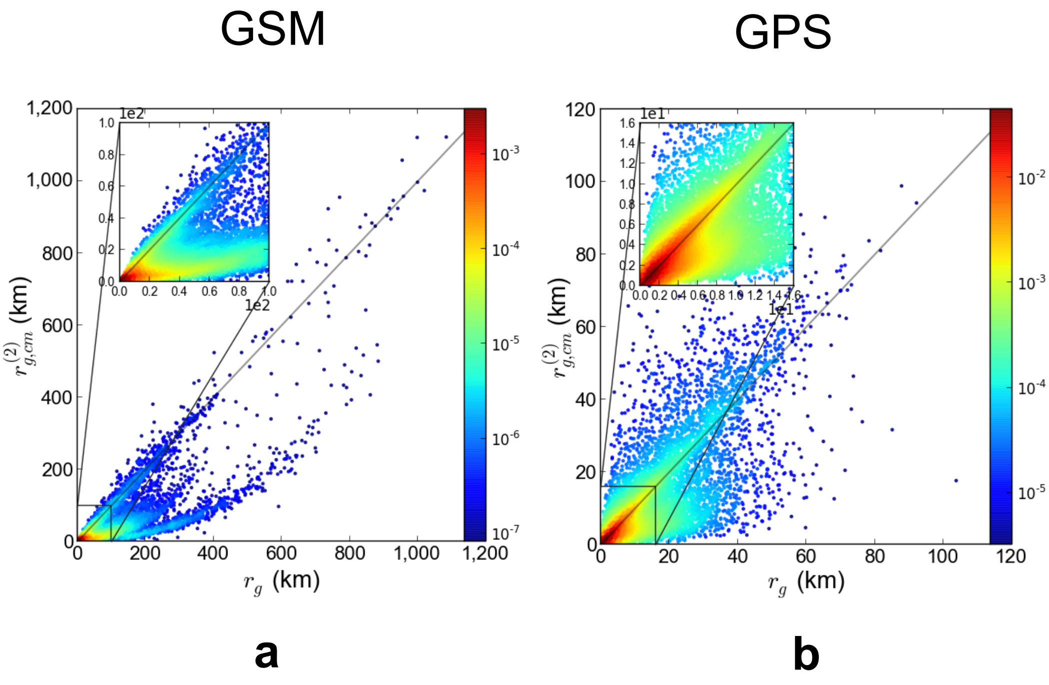

Supplementary Figure 8: **The correlation between total radius and k-radius computed on total center of mass.** Scatterplot of r_g versus $r_{g,cm}^{(k)}$ for GSM data (**a**) and GPS data (**b**), for $k = 2$. We observe that the split into 2-returners and 2-explorers is less clear for GSM data and absent for GPS data.

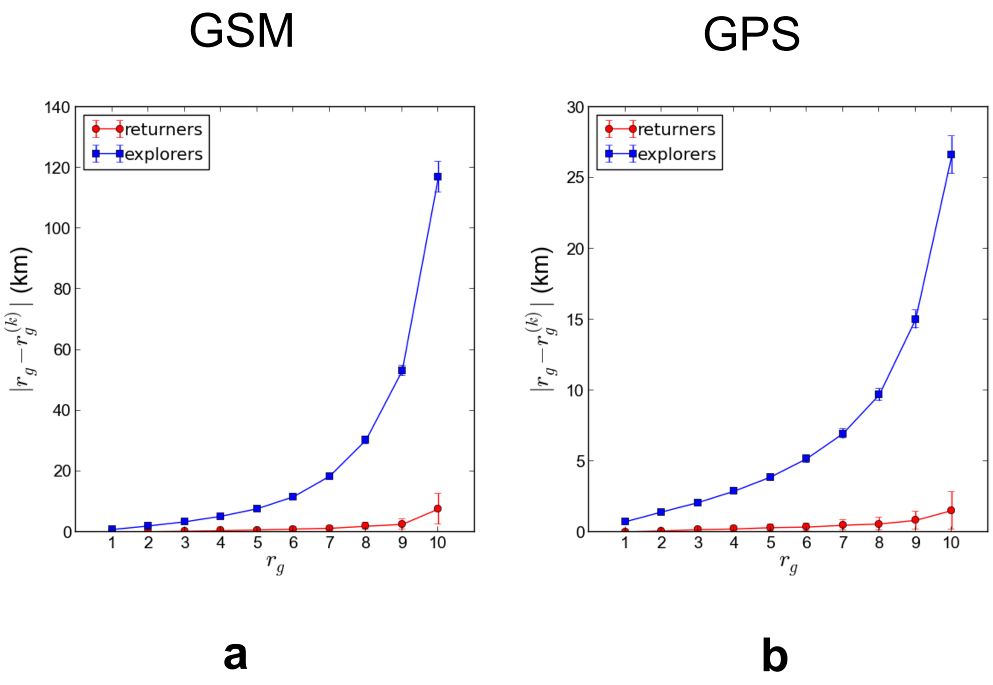

Supplementary Figure 9: **The distance between k-center of mass and total center of mass.** Distance between the 2-center of mass and the overall center of mass, relative to $r_g^{(2)}$: $(r_{cm} - r_{cm}^{(2)})/r_g^{(2)}$, where individuals are grouped according to the deciles of r_g.

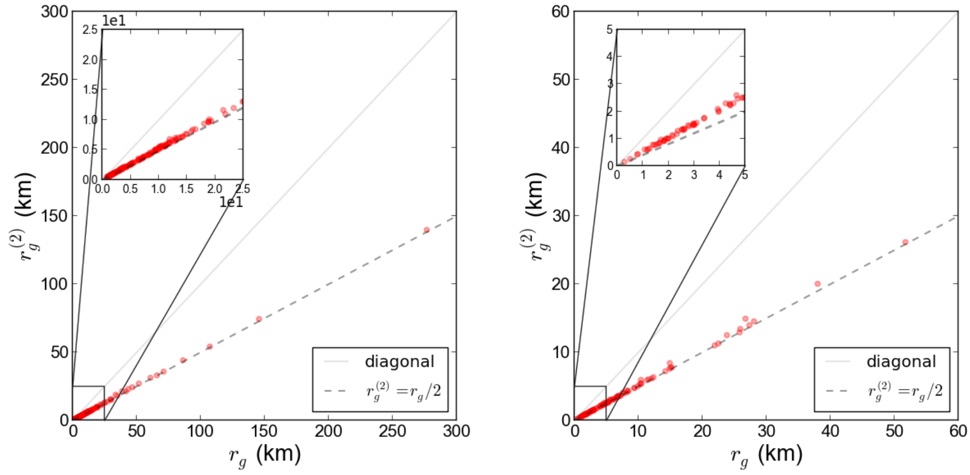

Supplementary Figure 10: **The transition from the returners state to the explorers state.** Scatterplot of r_g versus $r_g^{(2)}$ for GSM data (left) and GPS data (right). The dashed line indicates the curve $r_g^{(k)} = r_g/2$ which discriminates between 2-returners and 2-explorers according to the bisector method.

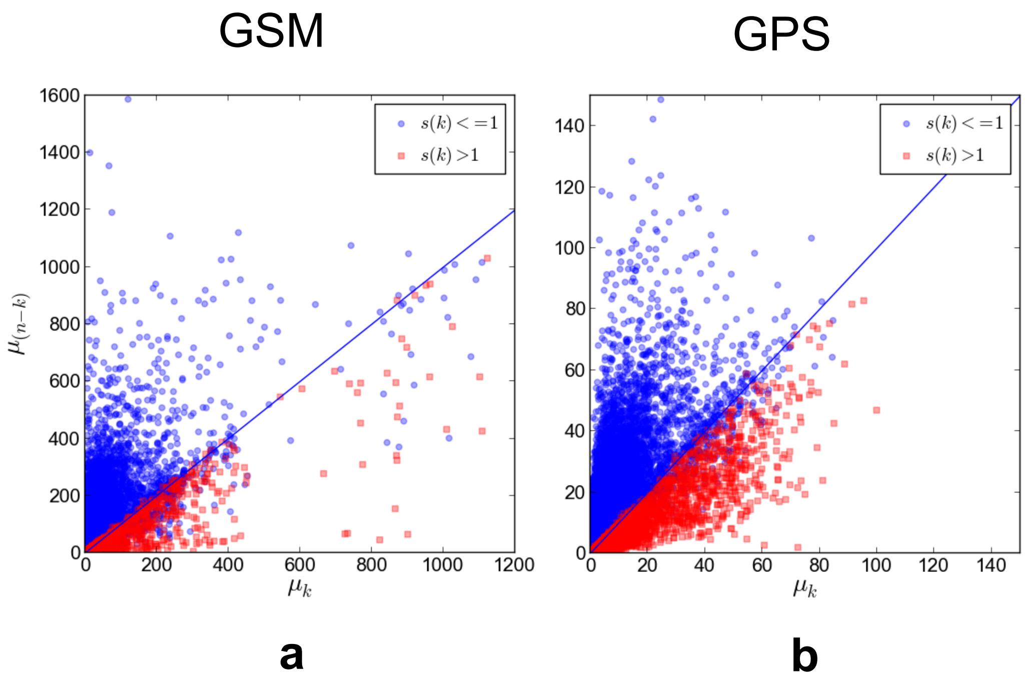

Supplementary Figure 11: **The correlation between μ_k and $\mu_{(n-k)}$.** Scatterplot of μ_k versus $\mu_{(n-k)}$ ($k = 2$) for GSM data (**a**) and GPS data (**b**). Red squares indicate individuals with $s_k = r_g^{(k)}/r_g > 1$, while the blue solid curve is the line $y = x$.

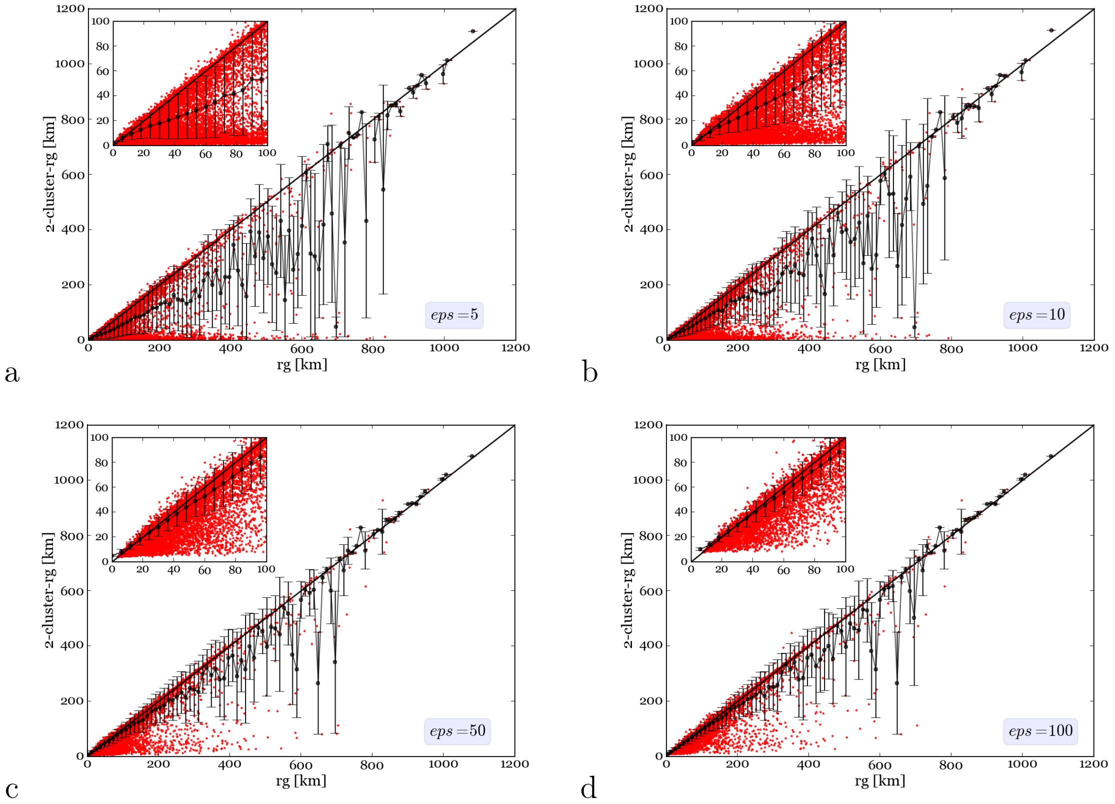

Supplementary Figure 12: **The returners/explorers dichotomy is independent of the geographic scale.** Scatterplots of r_g versus the cluster-$r_g^{(k)}$, for $k = 2$, GSM data. The geographic clusters are computed through the DBSCAN algorithm with parameters $eps = 5, 10, 50, 100$km and $minPts = 2$. The returner/explorer dichotomy appears again and it is clear until $eps = 10$km (**b**), where clusters have the size of medium-sized city.

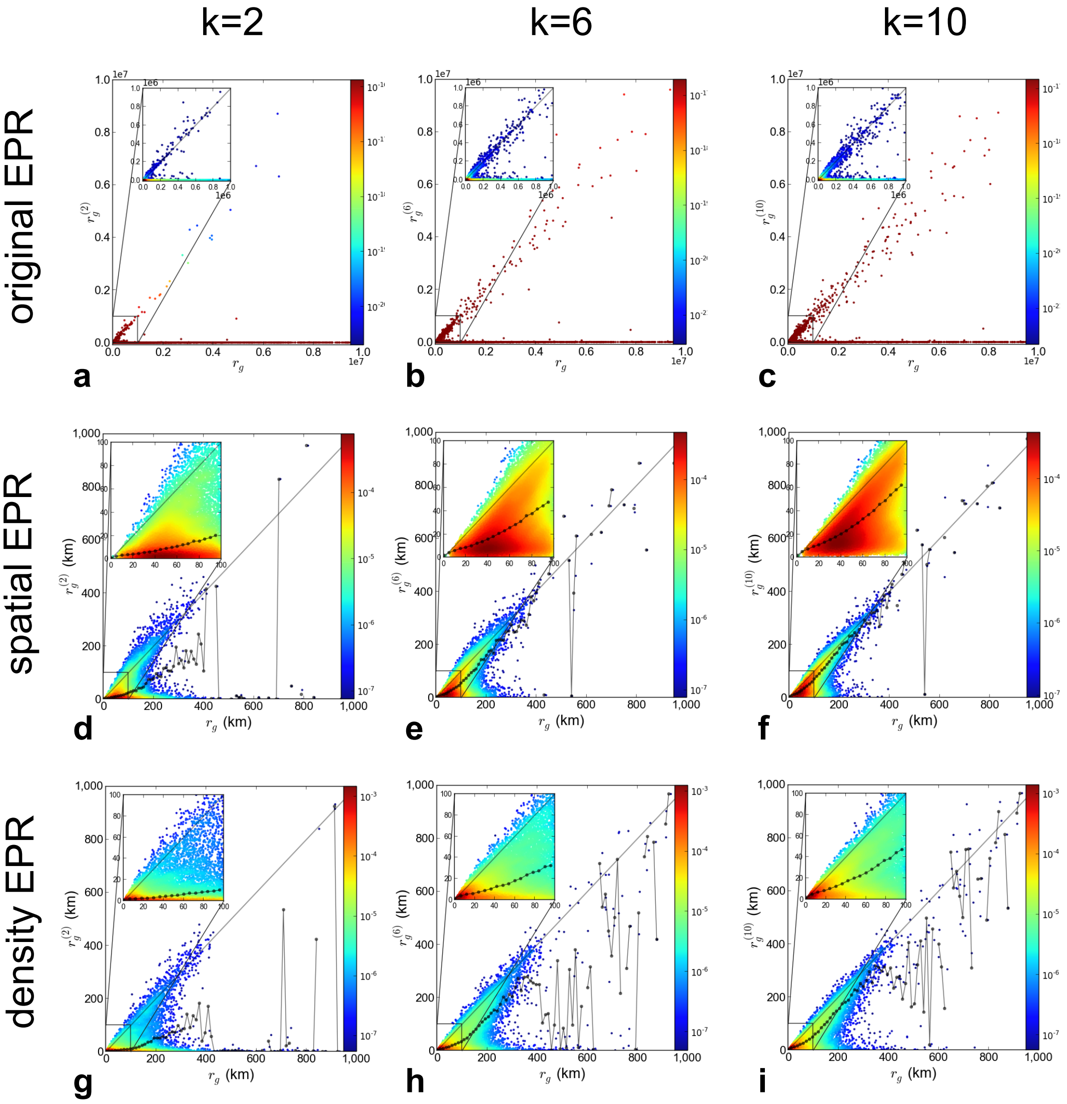

Supplementary Figure 13: **The correlation between total mobility and recurrent mobility according to mobility models.** The correlations between r_g and $r_g^{(k)}$ for $k = 2, 6, 10$ for the EPR dataset (**a, b, c**), the s-EPR dataset (**d, e, f**) and the d-EPR dataset (**g, h, i**).

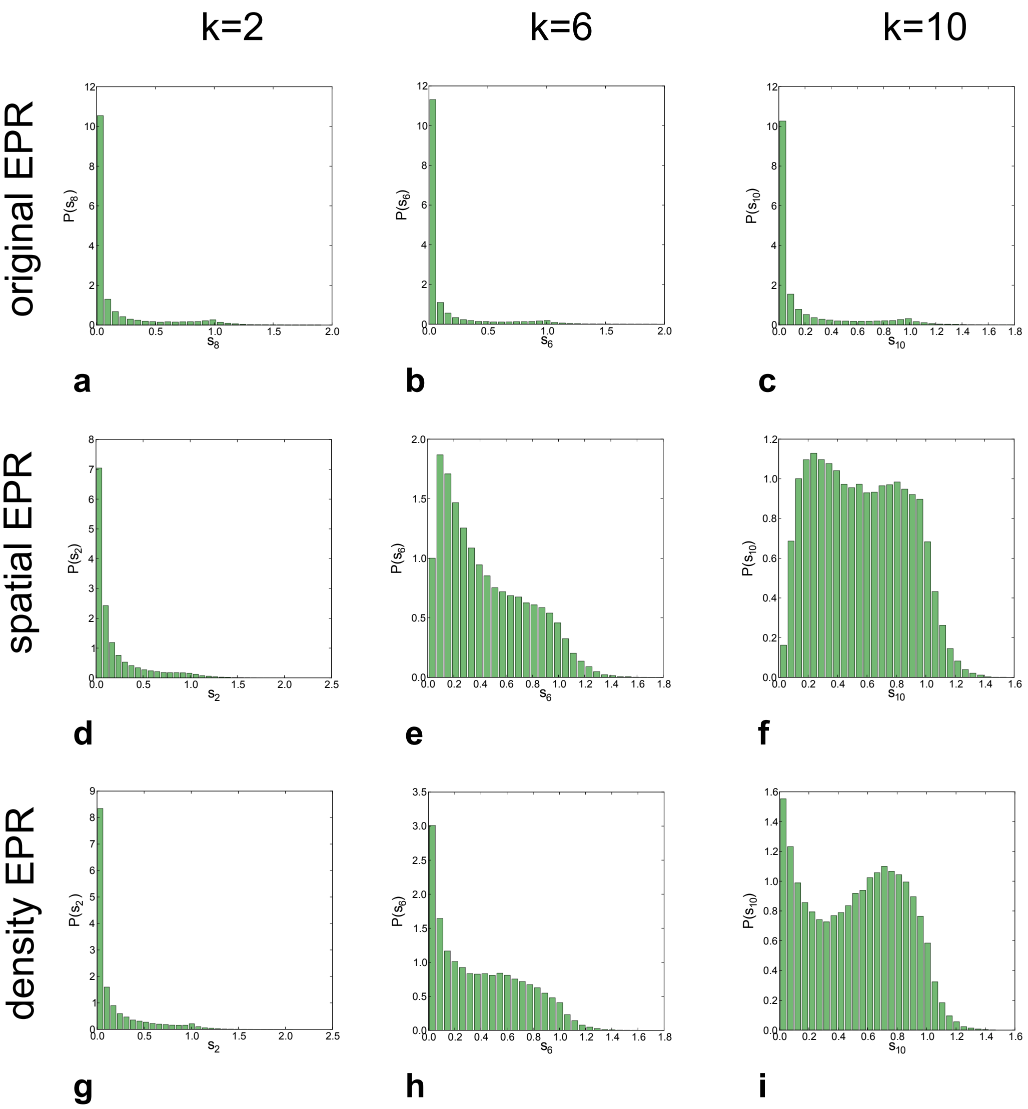

Supplementary Figure 14: **The ratio between recurrent mobility and total mobility according to the mobility models.** Distributions of the ratio $s_k = r_g^{(k)}/r_g$ with $k = 2, 6, 10$ for the original EPR dataset (**a, b, c**), the s-EPR dataset (**d, e, f**) and the d-EPR dataset (**g, h, i**).

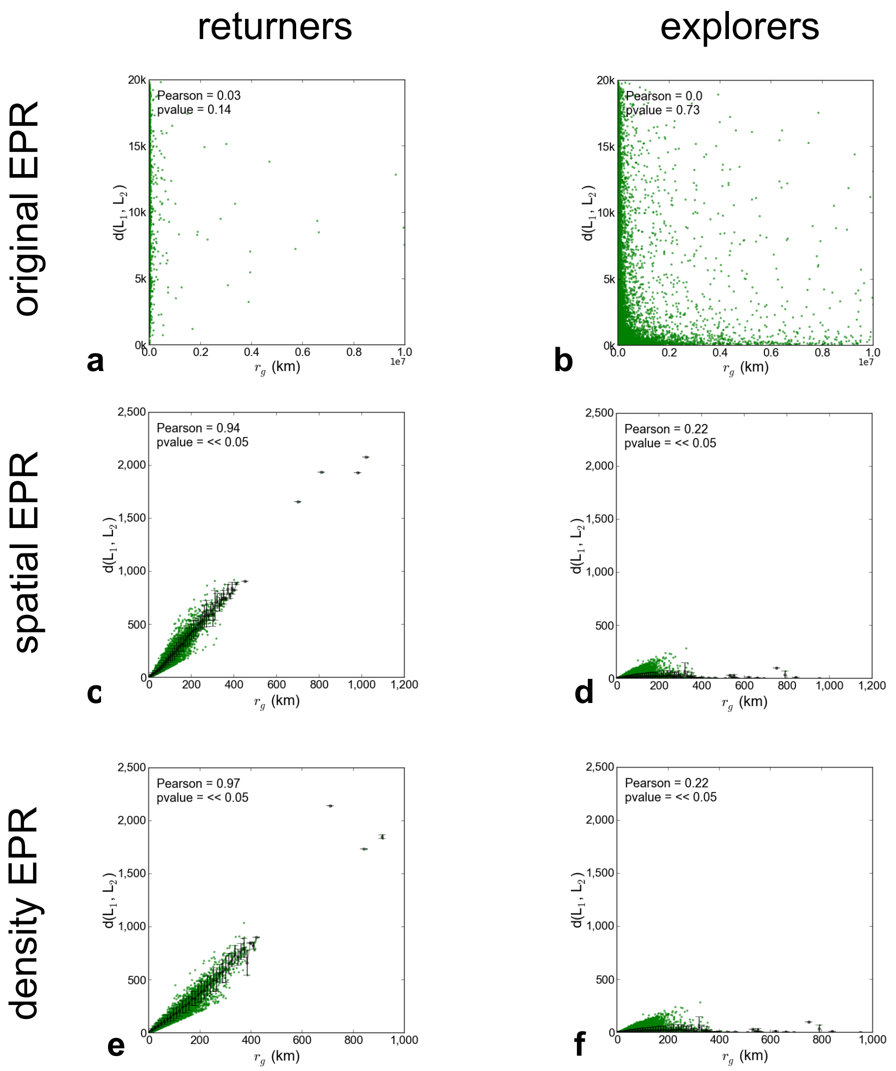

Supplementary Figure 15: **The correlation between total radius and the distance of the most frequented locations according to mobility models.** Correlation between the r_g and the distance between the two most frequent locations $dist(L_1, L_2)$ of 2-returners and 2-explorers, with $k = 2$, for the original EPR model (**a, b**), the s-EPR model (**c, d**) and the d-EPR model (**e, f**).

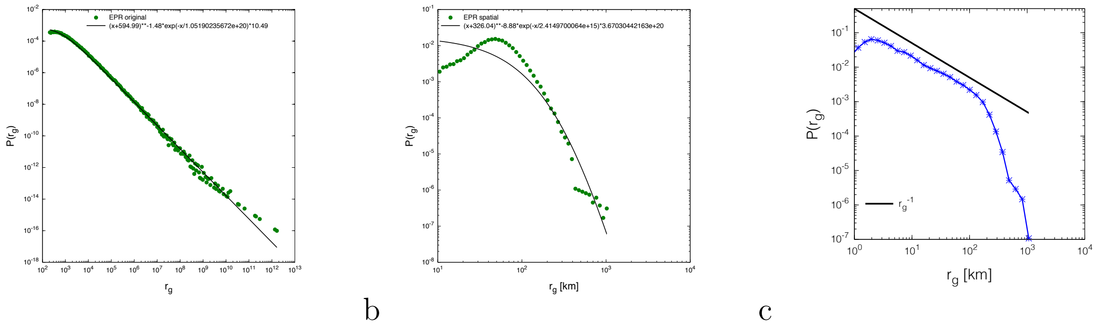

a b c

Supplementary Figure 16: **The distribution of total radius according to the mobility models.** The distribution of r_g for EPR model (**a**), s-EPR model (**b**), and d-EPR model (**c**).

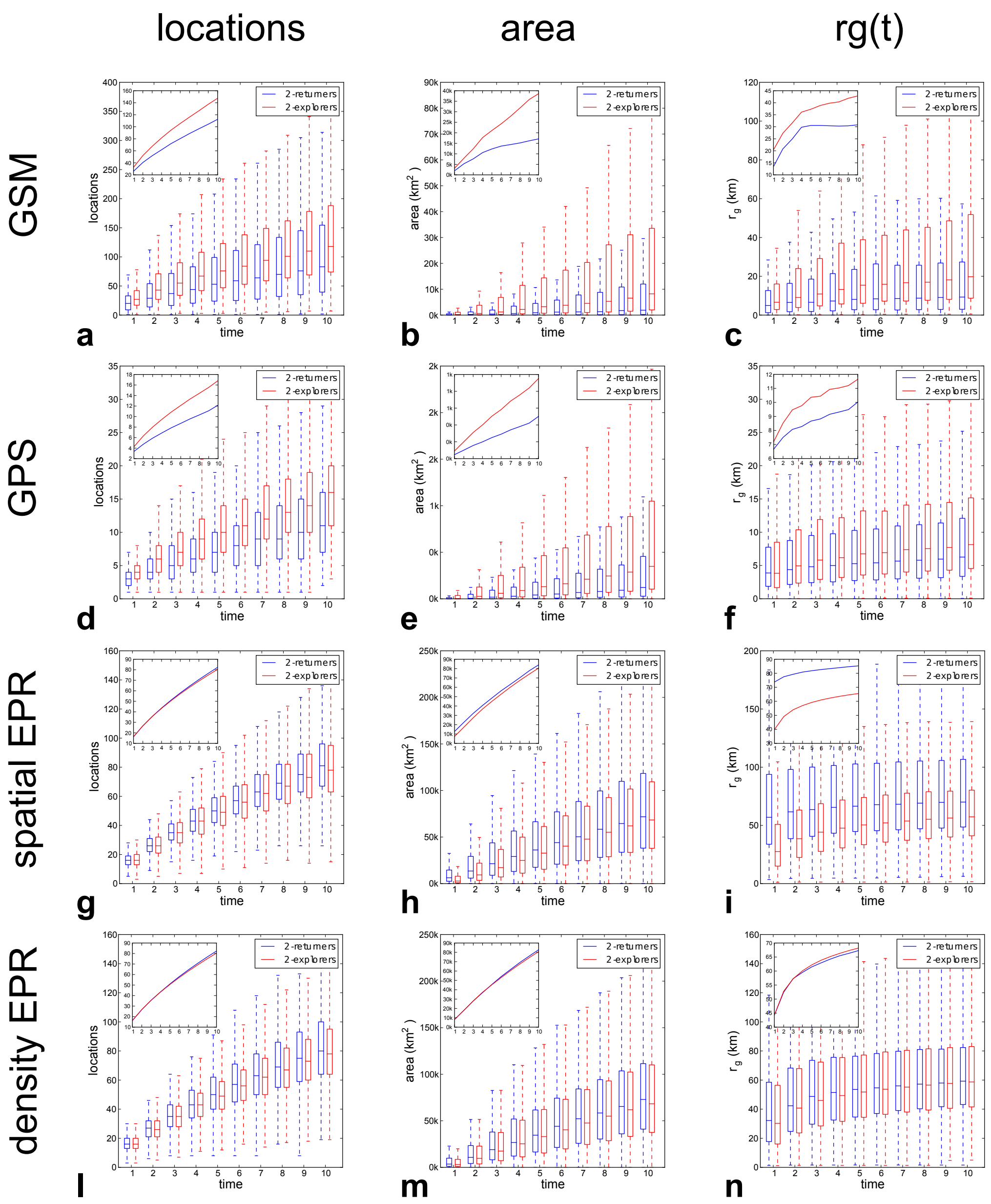

Supplementary Figure 17: **Temporal evolution of geographic spread for returners and explorers.** (**a**, **d**) The distribution of the number of location visited by 2-returners and 2-explorers. We split the mobility history of each individual into ten equal time slots. (**b**, **e**) The distributions of the area potentially covered by 2-returners and 2-explorers in each time slot. (**c**, **f**) The distributions of $r_g(t)$ for 2-returners and 2-explorers. (**g-i**) The three distributions for s-EPR and the d-EPR models. Models overestimate the geographical spread of 2-returners. The insets shows the mean of the distributions in each time slot.

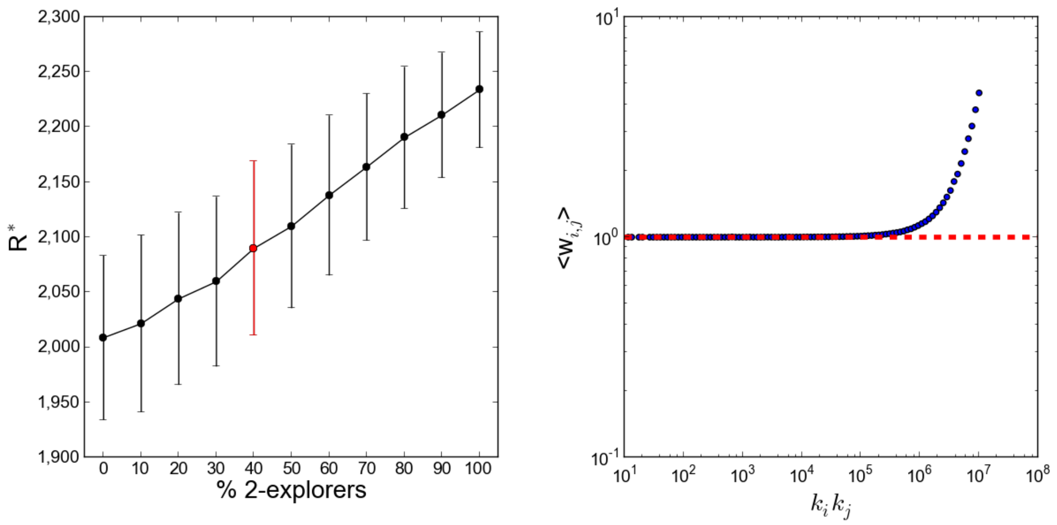

Supplementary Figure 18: **The global invasion diffusion threshold.** *(Left)* The error bars show how the distribution of the diffusion invasion threshold changes when different proportions of 2-returners and 2-explorers are chosen. The red error bar indicates the distribution where the fraction of explorers is 40%, the actual fraction of 2-explorers in GPS data according to the bisector method. *(Right)* Average weight as a function of the end-point degree. The dashed line corresponds to flat behavior ($\theta = 0$).

2 Supplementary Tables

timestamp	tower	caller	callee	type
2008/04/01 23:45:00	(132.567, 23.642)	A45J23	F45J23	SMS
2008/04/02 06:02:10	(143.282, 54.221)	K65232	V56YT4	Call
2008/04/02 06:15:12	(103.31, 22.34)	K65232	F45J23	Call
⋮	⋮	⋮	⋮	⋮

Supplementary Table 1: **Example of Call Detail Records (CDRs)**. Every time a user makes a call or sends an SMS a record is created with timestamp, tower serving the call, caller (anonymized) identifier, callee (anonymized) identifier and type of communication (SMS or call).

timestamp	origin	destination	vehicle
2011/05/12 08:31:20	(32.567, −2.546)	(32.7, −2.511)	F45J23
2011/05/24 17:53:08	(32.1982, −2.333)	(33.123, −2.31)	H2705L
2011/05/24 20:03:18	(33.15, −2.46)	(33.123, −2.31)	LP342L
⋮	⋮	⋮	⋮

Supplementary Table 2: **Example of GPS records.** Every time a vehicle stops for more than 20 minutes we store in the dataset the timestamp, the origin and destination, and the (anonymized) identifier of the vehicle.

3 Supplementary Notes

Supplementary Note 1: GSM data

The mobile phones carried by individuals in their daily routine offer a good proxy to study the structure and dynamics of human mobility: each time an individual makes a call the tower that communicates with her phone is recorded by the carrier, effectively tracking her current location. In our study we use an anonymized GSM dataset collected by a European carrier for billing and operational purposes. The dataset consists of Call Detail Records (CDR) describing each phone call performed by ≈3 million users in a period of three months. Each call is characterized by timestamp, caller and callee identifiers, duration of the call and the geographical coordinates of the tower serving the call (Supplementary Table 1). The time ordered list of towers from which a user made her calls forms a trajectory, capturing her movements during the period of observation [1].

We apply several filters to the data. Firstly, for each user u we discard all the locations with a visitation frequency $f = n_i/N \leq 0.005$, where n_i is the number of calls performed by u in location i and N the total number of calls performed by u during the period of observation. This condition checks whether the location is relevant with respect to the specific call volume of the user. Since it is meaningless to analyze the mobility of individuals who do not move, all the users with only one location after the previous filter are discarded. We select only active users with a call frequency threshold of $f = N/(24*91) \geq 0.5$, where N is the total number of calls made by u, 24 is the hours in a day and 91 the days in our period of observation. Finally, to exclude abnormally active users like line testers and alarm managers we discard the users with a huge number of calls $N > k * 91$, where $k = 300$. Starting from ≈3 millions users, the filtering results in $67,049$ active mobile phone users.

Supplementary Note 2: GPS data

The GPS dataset stores information of approximately 9.8 Million different trips from 159,000 vehicles tracked during one month (May 2011) which passed through a 250km×250km square in central Italy. The GPS traces are provided by Octo Telematics Italia Srl (`http://www.octotelematics.com/`), a company that provides a data collection service for insurance companies. The market penetration of this service is variable on the territory, but covers in average around 2% of the total registered vehicles. The GPS device automatically turns on when the vehicle starts, and the sequence of GPS points that the device transmits every 30 seconds to the server via a GPRS connection forms the global trajectory of a vehicle. When the vehicle stops no points are logged nor sent. We exploit these stops to split the global trajectory into several sub-trajectories, corresponding to the trips performed by the vehicle. Clearly, the vehicle may have stops of different duration, corresponding to different activities. To ignore small stops like gas stations, traffic lights, bring and get

activities and so on, we choose a stop duration threshold of at least 20 minutes: if the time interval between two consecutive observations of the vehicle is larger than 20 minutes, the first observation is considered as the end of a trip and the second observation is considered as the start of another trip. We also performed the extraction of the trips by using different stop duration thresholds (5, 10, 15, 20, 30, 40 minutes), without finding significant differences in the sample of short trips and in the statistical analysis we present in the paper. Since GPS data do not provide explicit information about visited locations, we assigned each origin and destination point of the obtained sub-trajectories to the corresponding census cell, according to the information provided by the Italian National Institute of Statistics (ISTAT, `www.istat.it`). As for the GSM data, we describe the movements of a vehicle by the time-ordered list of census cells where the vehicle stopped (Supplementary Table 2). We filter the data by focusing only on trips performed within a single region (Tuscany), and by discarding all the vehicles with only one visited location or with less than one trip per day on average during the period of observation. This filtering results in a dataset of 46,121 vehicles.

The GSM and the GPS datasets differ in several aspects [2, 3]. The GPS data refers to trips performed during one month (May 2011) in an area corresponding to a single Italian region, while the mobile phone data cover an entire European country and a period of observation of three months. The GPS data represents a 2% sample of the population of vehicles in Italy [2], while the mobile phone dataset covers users of a major European operator, about the 25% of the country's adult population. The trajectories described by mobile phone data include all possible means of transportation. In contrast, the GPS data refers to vehicle displacements only. The fact that one dataset contains aspect missing in the other dataset makes the two types of data suitable for an independent validation of the universality of the patterns emerging from human mobility behavior.

Supplementary Note 3: importance of locations

We evaluate the importance of a GSM location, i.e. its weight, using the visitation frequency as suggested in the seminal paper by González et al. [1]. For GPS data, we measure the weight using the dwell time of a vehicle in a certain census cell. As Supplementary Figure 1 suggests, while in the GSM data there is no significant difference between the frequency and time distribution, for the GPS data the time spent in a location is much more discriminant of the importance of a location.

Supplementary Note 4: classification methods

We develop three methods to split the population into returners and explorers. The bisector method uses a curve bisecting the plane to detect the subpopulation of k-returners. A Support Vector Machine (SVM) and the Expectation-Maximization (EM) clustering algorithm extract the two patterns from the population by means of data mining techniques.

The bisector method uses the curve $r_g^{(k)} - r_g/2 = 0$ to bisect the plane, defining all the users above the curve as k-returners. Supplementary Figure 3 shows how the number of k-returners varies with the number of locations k considered into the k-radius. Supplementary Figure 2(a, d) shows the split of the population according to the bisector method.

Support Vector Machines (SVM) [4] are supervised learning models that analyze data and recognize patterns. We first build the SVM classifier providing a set of training examples to the SVM learning algorithm, and then used the built model to classify individuals as k-returners or k-explorers. We describe each individual as a pair $(r_g^{(k)}, r_g)$. As training examples, we select the individual falling exactly on the diagonal (k-returners) or the abscissa (k-returners) of the r_g vs $r_g^{(k)}$ plot. Precisely, k-returners examples are all the individual for which $r_g^{(k)} = r_g$, while k-explorer examples are all the individual for which $r_g^{(k)} = 0$. Supplementary Figure 2(b, e) shows the split of the population according to the SVM method.

The Expectation-Maximization (EM) algorithm [4] is an iterative method for finding maximum likelihood of parameters in statistical models. It alternates between an expectation (E) step, which creates a function for the expectation of the log-likelihood based on the current estimate for the parameters, and a maximization (M) step, which computes parameters maximizing the expected log-likelihood found on the E step. These parameter-estimates are then used to determine the distribution of the latent variables in the next E step. The EM algorithm outputs a pair of values for each individual, representing the probability to be a k-returner and a k-explorer. We assign each individual to the category with the highest probability. Supplementary Figure 2(c, f) shows the split of the population according to the EM method.

The three methods produce similar trends of variation with k (Supplementary Figure 3). For GSM data, k-returners are initially the minority in the population. They start outnumbering the k-explorers from $k = 4$. In the GPS case k-returners are immediately the majority, and the gap increases with the value of k. Since the methods produce similar results, we focus on the simplest, the bisector method.

Supplementary Note 5: spatial distribution of locations

The spatial distribution of the visited locations is a characteristic feature that differentiates between returners and explorers. We show that we can provide a quantitative and robust confirmation to this observation by presenting the following two results:

1. The distance between the two most frequented locations of 2-returners grows proportionally to their total radius of gyration, while it is not so for 2-explorers.

2. The locations visited by 2-returners are clustered around their two most frequented locations, while those visited by 2-explorers are more spread out.

To show the validity of (1) we plot the correlation between r_g and the distance between the $k = 2$ most frequented locations, separately for returners and explorers. We observe that the positive correlation between r_g and the distance $dist(L_1, L_2)$ is stronger for 2-returners than 2-explorers, both for GSM data (Supplementary Figure 4 a, b) and GPS data (Supplementary Figure 4 c, d). Therefore for returners there is a tendency of the k most frequent locations to move far away from each other as total r_g increases. This tendency is very weak for explorers.

To show the validity of (2) we compute the clusters around the k most frequented locations in the following way: the k most frequented locations $L_1, \dots L_k$ are the centroids of k different clusters $C_1, \dots, C_k$, then we assign each location L_i $(i > k)$ to the cluster of the closest centroid, i.e. if L_i is closer to L_1 than the other $k - 1$ most frequented locations we assign it to cluster C_1. For each user we evaluate the cohesion of its clusters using two measures: the SSE and the SSE*. The SSE (Sum of Squared Errors) is the total sum of the squared distances of locations within a cluster to their centroid, $SSE = \sum_{i=1}^{k} \sum_{x \in C_i} dist(c_i, x)^2$ where c_i is the centroid of cluster C_i. The higher the SSE, the worse is the cohesion of the clusters. The SSE* is another measure of cluster cohesion: $SSE^* = SSE/\overline{SSE}$, where $\overline{SSE} = \sum_{i=1}^{k} \sum_{x \in C_i} \sum_{j=1, j \neq k}^{k} dist(c_j, x)^2$ is the sum of squared distances of each location to the centroids of the other clusters. Supplementary Figure 5 shows the distribution of SSE and SSE* separately for 2-returners and 2-explorers and for GSM and GPS data. We observe that a large fraction of 2-returners have dense clusters around the 2 most important locations (small SSE and SSE*), while 2-explorers have lower cohesion, on average.

We also investigate the correlation between $d(L_1, L_2)$ and SSE of individuals, observing that 2-returners and 2-explorers follow two distinct behaviors. The SSE for 2-explorers assumes values far larger than 2-returners, while the distance $d(L_1, L_2)$ for 2-returners has far larger variability than 2-explorers (Supplementary Figure 6). For 2-returners the distance $d(L_1, L_2)$ significantly changes with the radius of gyration while for 2-explorers it does not, and the k clusters are much more cohesive for 2-returners than 2-explorers.

Supplementary Note 6: call activity and demography variables

We have verified that the split of individuals into returners and explorers is not due to confounding variables like 1) the heterogeneity on the number of calls, and 2) the demography of the municipality of residence.

1. In Supplementary Figure 7 (left) we show the two probability density functions of the total number of calls made by 2-returners (blue solid curve) and 2-explorers (red dashed curve). The two curves are very close, confirming that the level of activity of the individuals in the two groups is comparable and thus excluding a possible bias due to heterogeneous call frequencies.

2. In Supplementary Figure 7 (right) we consider groups of municipalities with similar population and compute the fraction of 2-returners living in those municipalities. From the figure it is clear that the fraction of 2-returners in a municipality is (i) independent of the population of the municipality, and (ii) compatible (within a standard deviation) with the overall fraction of 2-returners in the country. Hence, we can exclude that the demography of the municipality of residence may affect the classification of individuals to the observed groups of mobility behaviours.

Supplementary Note 7: k-radius on total center of mass

We also compute for each individual $r_{g,cm}^{(k)}$ which is the k-radius computed using the overall center of mass instead of $r_{cm}^{(k)}$. In the scatterplot r_g vs $r_{g,cm}^{(k)}$ the split into 2-returners and 2-explorers is less clear for GSM data, and it is absent for GPS data (Supplementary Figure 8). Explorers move towards the diagonal ($r_{g,cm}^{(k)} \geq r_g^{(k)}$) suggesting that for explorers $r_{cm}^{(k)}$ is different from r_{cm} (Supplementary Figure 8). Supplementary Figure 9 shows the error bars of the distance $r_{cm} - r_{cm}^{(k)}$ relative to $r_g^{(k)}$, where individuals are grouped according to the deciles of r_g. While the relative distance is constant for 2-returners (the two centers of mass are relatively close to each other), for 2-explorers the relative distance is higher and increases with r_g. As a consequence, 2-returners have similar $r_g^{(k)}$ and $r_{g,cm}^{(k)}$ while this is not true for 2-explorers.

Supplementary Note 8: transition between the two states

While explorers gradually become returners as k increases, the opposite process is extremely rare. For $k = 3, \ldots 10$ we compute the fraction $\bar{n}$ of users who are k-explorers and $(k-1)$-returners. The number $\bar{n}$ decreases with k and is very small, lower than 0.01 for any k. In particular for $k = 3$, $\bar{n} = 0.008$ for GSM data (185 individuals) and $\bar{n} = 0.004$ for GPS data (105 individuals). We plot the correlation between r_g and $r_g^{(2)}$ for these individuals and observe that they are located on the curve $r_g^{(k)} = r_g/2$ (Supplementary Figure 10). Since they are on the bisector line, they are not 2-returners neither 2-explorers the bisector

method fails in classifying properly the individuals and generates the fluctuations between the two profiles as k increases.

Supplementary Note 9: distance of locations to the center of mass

The ratio $s_k = r_g^{(k)}/r_g$ is less than one for almost all individuals. $s_k > 1$ means that $r_g^{(k)} > r_g$ suggesting that the $(n-k)$ less frequented locations are on average closer to the center of mass than the k most frequented locations. We verify this hypothesis by computing two measures:

- $\mu_k = 1/k \sum_{i=1}^{k}(r_i - r_{cm})$, the mean distance of the k most frequented locations to the center of mass;
- $\mu_{(n-k)} = 1/(n-k) \sum_{i=k+1}^{n}(r_i - r_{cm})$, the mean distance of the other $n-k$ locations to the center of mass.

Supplementary Figure 11 shows the scatterplot of μ_k versus $\mu_{(n-k)}$ for $k = 2$. We observe that individuals with $s_k > 1$ are below the bisector of the plane meaning that $\mu_k \geq \mu_{(n-k)}$. Hence for individuals with $r_g^{(k)} > r_g$ the k most frequented locations are distant from the center of mass while the other $n-k$ are very close contributing the produce a total r_g smaller than $r_g^{(k)}$.

Supplementary Note 10: mobility clusters

We define the *cluster k-radius* $c\text{-}r_g^{(k)}$ as the radius of gyration computed on the k most frequented geographic clusters. A geographic cluster of an individual is a dense group of locations representing a geographic unit of individual mobility. An individual that commutes weekly between two homes in two different cities has (at least) two different geographic clusters. The $c\text{-}r_g^{(k)}$ is computed on the k most frequented clusters, considering the most frequent location of each cluster only. In the above cited example, the cluster radius of an individual is the radius of gyration computed on the two different homes.

We compute the geographical clusters through the DBSCAN algorithm [4], which extracts dense groups of points according to two input parameters: *eps*, the maximum search radius; and *minPts*, the minimum number of points (locations) to form a cluster. We set *minPts* $= 2$ and *eps* $= 5, 10, 50, 100$km. The split into 2-returners and 2-explorers emerges also at cluster level, and it is clear until *eps* $= 10$km, where the clusters have the size of a medium sized city. For high values of *eps* $= 50, 100$km the number of computed clusters is small (mainly 2), penalizing the presence of 2-explorers (Supplementary Figure 12). The presence of returners and explorers in the population, hence, is independent of the spatial granularity of individuals' location: it appears for GSM towers as well as when we take districts or entire cities as individual locations.

Supplementary Note 11: comparison with mobility models

We compare our findings with the results produced by the Exploration and Preferential Return (EPR) individual mobility model [5], a state-of-the-art model that accurately captures the visitation frequency of locations, the distribution of the radius of gyration across the population and its growth with time. The model does not fix the set of preferred locations but allows them to emerge naturally during the evolution of the mobility process. It incorporates two competing mechanisms: exploration and preferential return. Exploration is a random walk process with truncated power law jump size distribution. Preferential return reproduces the propensity of humans to return to the locations they visited frequently before. An agent in the model selects between the two modes: with probability $P_{new} = \rho S^{-\gamma}$ (where S is the number of locations visited so far by the agent, ρ and γ are two model parameters), the individual moves to a new location, whose distance from the current one is chosen from the known power law distribution of displacements. With complementary probability $P_{new} = 1 - \rho S^{-\gamma}$, the agent returns to one of the S previously visited places (with the preference for a location proportional to the frequency of visits). As a result, the model has a warmup period of greedy exploration, while in the long run agents mainly move around a set of previously visited places.

We implemented the original version of the EPR model, along with two improved versions: the s-EPR model, where agents are constrained within a limited geographical space; and the d-EPR model, in which an individual selects a new location depending on both its distance from the current position and its relevance measures ad the overall number of calls places by all users from that location. We use the gravity model to assign the probability of a trip between any two locations automatically constraining individuals within the country's boundaries. Supplementary Figures 13, 14 and 15 show the patterns of returners and explorers emerging from the three versions of the EPR model.

Supplementary Note 12: EPR model

Here we describe the implementation of the original EPR model. We generate an initial (home) location for each of the 67,000 synthetic individuals by randomly selecting a point on a square of size 100×100. We then repeat the following steps 1,000 times for each individual:

1. We extract a waiting time Δt from the distribution $P(\Delta t) \sim \Delta t^{-1-\beta} \exp^{(-\Delta t/\tau)}$, with $\beta = 0.8$ and $\tau = 17$ hours as measured in [5].

2. With probability $P_{new} = \rho S^{-\gamma}$, where S is the number of distinct locations previously visited and $\rho = 0.6$ and $\gamma = 0.21$ [5], the individual visits a new location (step 3), otherwise she returns to a previously visited location (step 4).

3. If the individual explores a new location, a distance Δr is extracted from the distribution $P(\Delta r) = \Delta r^{-1-\alpha}$ with $\alpha = 0.55$ as in [5], and the individual moves to a randomly selected location on the circle of radius Δr centered on her current location. The number of distinct locations visited, S, is increased by one. The new locations can be outside the initial 100×100 square.

4. If the individual returns to a previously visited location, it is chosen with probability proportional to the number of visits to that location.

Supplementary Note 13: spatial EPR model

Here we describe the implementation of the s-EPR model, a version of the EPR model where we constraint individual within spatial boundaries. We place each of the $67,000$ GSM users in her most visited location (GSM cell phone towers). For each individual we repeat the following steps:

1. *Same as the original model.*

2. *Same as the original model.*

3. If the individual explores a new location, a distance Δr is extracted from the distribution $P(\Delta r) = \Delta r^{-1-\alpha}$ with $\alpha = 0.55$ as in [5], and an angle θ between 0 and 2π is extracted with uniform probability. If the location at distance Δr and angle θ from the current location is not in the country's boundaries a new distance and a new angle are extracted until this condition is satisfied. When the new location is found the number of distinct locations visited, S, is increased by one.

4. *Same as the original model.*

Supplementary Note 14: density EPR model

Here we describe the implementation of the d-EPR model. We place each of the $67,000$ GSM users in their most visited location (GSM cell phone towers). For each individual we repeat the following steps:

1. *Same as the original model.*

2. *Same as the original model.*

3. If the individual who is currently in location i explores a new location, then the new location $j \neq i$ is selected according to the gravity model [6, 7] with probability $p_{ij} = \frac{1}{N}\frac{n_i n_j}{r_{ij}^2}$, where $n_{i(j)}$ is the total number of calls placed by all users from location $i(j)$ representing its relevance, r_{ij} is the geographic distance between i and j, and $N = \sum_{i,j\neq i} p_{ij}$ is a normalisation constant. The number of distinct locations visited, S, is increased by one.

4. *Same as the original model.*

Supplementary Note 15: temporal evolution of geographic spread

Following the temporal evolution of an individual's trajectory, we split her mobility history into time periods, and capture the geographical spread up to time t through the number of locations visited, the area covered and the radius of gyration $r_g(t)$. Explorers distribute over a larger territory, as they visit more locations, cover a lager geographic are and have a higher $r_g(t)$ with respect to returners (Supplementary Figure 16). In contrast, the s-EPR model and the d-EPR model overestimate the geographical spread of returners.

Supplementary Note 16: global diffusion invasion threshold

We compute the diffusion invasion threshold on several (unweighted) global mobility networks built by choosing randomly both 2-returners and 2-explorers with different proportions, in order to understand how the threshold changes as the fraction of explorers in the population increases. For each network we compute the mean degree $\langle k\rangle$, the mean square degree $\langle k^2\rangle$, and the mean number of residents in the each location $\bar{N}$. We use these values to determine the global invasion threshold $R_* = \bar{N}\cdot C(\langle k^2\rangle - \langle k\rangle)/\langle k\rangle^2$, under the assumption of a diffusion dynamics with large subpopulations and a low reproductive number (i.e. close to the subpopulation epidemic threshold) [8]. Here the constant C depends on the disease model and the mobility parameters (e.g. the reproductive number and the mobility rate), which are the same for the two classes of user profiles. In a metapopulation network an epidemic can spread and invade the system only if $R_* > 1$, and this global invasion threshold is affected by the topological fluctuations of the network's degree: the larger is the degree heterogeneity, the higher is R_* and therefore the higher is the chance

that the epidemic will globally invade the metapopulation. Error bars in Supplementary Figure 17 (left) summarize the distribution (mean and standard deviation) of the diffusion invasion threshold over 1,000 random experiments in ten different scenarios where different proportions of 2-returners and 2-explorers are chosen. We observe that as the fraction of 2-explorers increases the mean diffusion invasion threshold increases.

We also build weighted global mobility networks using the number of trips between locations performed by vehicles during the period of observation as weight of the edges. The distribution of edge weights follows a power law, with maximum values of 30 (i.e. 30 trips between the locations). This is presumably due to the size of the census cells, which is not uniform and tend to be very small in densely populated areas. To compute a weighted version of the global invasion threshold $R_*^w = \bar{N} \cdot (\langle k^{2+2\theta} \rangle - \langle k^{1+2\theta} \rangle)/\langle k^{1+\theta} \rangle^2$ we estimate the parameter θ as the function between the average weight and the end-point degrees [9, 10] (Supplementary Figure 17, right). We observe a flat behavior for almost six orders of magnitude, hence we estimate $\theta = 0$. For $\theta = 0$ the $R_*^w = R_*$ being undistinguishable to the unweighted scenario.

Supplementary References

[1] González, M. C., Hidalgo, C. A. & Barabási, A.-L. Understanding individual human mobility patterns. *Nature* **453**, 779–782 (2008).

[2] Pappalardo, L., Rinzivillo, S., Qu, Z., Pedreschi, D. & Giannotti, F. Understanding the patterns of car travel. *The European Physical Journal Special Topics* **215**, 61–73 (2013).

[3] Pappalardo, L., Simini, F., Rinzivillo, S., Pedreschi, D. & Giannotti, F. Comparing general mobility and mobility by car. In *Proceedings of the 1st BRICS Countries Congress (BRICS-CCI) and 11th Brazilian Congress (CBIC) on Computational Intelligence* (2013).

[4] Tan, P.-N., Steinbach, M. & Kumar, V. *Introduction to Data Mining* (Addison Wesley, 2006).

[5] Song, C., Koren, T., Wang, P. & Barabási, A.-L. Modelling the scaling properties of human mobility. *Nature Physics* **6**, 818–823 (2010).

[6] Zipf, G. K. The p1p2/d hypothesis: On the intercity movement of persons. *American Sociological Review* **11**, 677–686 (1946).

[7] Jung, W. S., Wang, F. & Stanley, H. E. Gravity model in the korean highway. *EPL (Europhysics Letters)* **81**, 48005 (2008).

[8] Colizza, V. & Vespignani, A. Invasion threshold in heterogeneous metapopulation networks. *Physical Review Letters* **99**, 148701 (2007).

[9] Colizza, V. & Vespignani, A. Epidemic modeling in metapopulation systems with heterogeneous coupling pattern: Theory and simulations. *Journal of Theoretical Biology* **251**, 450–467 (2008).

[10] Barrat, A., Barthélemy, M., Pastor-Satorras, R. & Vespignani, A. The architecture of complex weighted networks. *Proceedings of the National Academy of Sciences of the United States of America (PNAS)* **101**, 3747–3752 (2004).

Correspondence and requests for materials should be addressed to: lpappalardo@di.unipi.it and f.simini@bristol.ac.uk
